# Highly Invasive Fluorescent/Bioluminescent Patient-Derived Orthotopic Model of Glioblastoma in Mice

**DOI:** 10.3389/fonc.2022.897839

**Published:** 2022-07-13

**Authors:** Diana Yuzhakova, Elena Kiseleva, Marina Shirmanova, Vladislav Shcheslavskiy, Daria Sachkova, Ludmila Snopova, Evgeniya Bederina, Maria Lukina, Varvara Dudenkova, Gaukhar Yusubalieva, Tatyana Belovezhets, Daria Matvienko, Vladimir Baklaushev

**Affiliations:** ^1^ Institute of Experimental Oncology and Biomedical Technologies, Privolzhsky Research Medical University, Nizhny Novgorod, Russia; ^2^ R&D Department, Becker&Hickl GmbH, Berlin, Germany; ^3^ Institute of Biology and Biomedicine, Lobachevsky State University of Nizhny Novgorod, Nizhny Novgorod, Russia; ^4^ Laboratory of Molecular Oncology, Federal Research and Clinical Center of Physical and Chemical Medicine, Moscow, Russia; ^5^ Biomedical Research Center, Federal Research and Clinical Center, Federal Medical and Biological Agency, Moscow, Russia; ^6^ Laboratory of Molecular Mechanisms of Regeneration and Aging, Engelhardt Institute of Molecular Biology, Moscow, Russia; ^7^ Department of Molecular Immunology, Institute of Molecular and Cellular Biology SB RAS, Novosibirsk, Russia

**Keywords:** glioblastoma (GBM), primary cell line, patient-derived xenograft (PDX), fluorescence imaging, FLIM (fluorescence lifetime imaging microscopy)

## Abstract

Development of the novel diagnostic and therapeutic approaches in neuro-oncology requires tumor models that closely reproduce the biological features of patients’ tumors. Patient-derived xenografts (PDXs) are recognized as a valuable and the most “close-to-patient” tool for preclinical studies. However, their establishment is complicated by the factors related to both the surgical material and technique of the orthotopic implantation. The aim of this work was to develop a patient-derived glioblastoma multiform (GBM) model that stably co-expresses luciferase and a far-red fluorescent protein for monitoring of tumor progression in the brain and, using this model, to validate new diagnostic methods—macroscopic fluorescence lifetime imaging (macro-FLIM) and cross-polarization optical coherence tomography (CP OCT). The established model was similar to the original patient’s GBM in terms of histological and immunohistochemical features and possessed reproducible growth in nude mice, which could be observed by both fluorescence and bioluminescence imaging. Our results demonstrated the high potential of macro-FLIM and CP OCT for intraoperative differentiation of GBM from the white matter. Thus, the dual-labeled PDX model of GBM proved to be an excellent approach for observation of tumor development by optical methods.

## Introduction

GBM (glioma grade IV) is the most common brain malignancy in adults, representing approximately 57% of all gliomas and 48% of all primary malignant tumors of the central nervous system (CNS). Current standard of care for GBM includes maximal surgical resection, followed by radiotherapy and temozolomide chemotherapy (1). Unfortunately, GBM recurrence is inevitable, and prognosis for patients is very poor—the 5-year survival rate of adult patients is around 4.3% (2). Two features of GBM greatly complicate the therapy: 1) its aggressive growth with diffuse invasion into healthy brain tissue and 2) significant intra-tumoral heterogeneity of malignant cells.

Development of new diagnostic and therapeutic strategies requires animal models that would accurately mimic human GBMs (3, 4). An ideal human GBM model should possess the following characteristics: 1) be highly reproducible with predictable growth in mice; 2) be orthotopic to provide the appropriate microenvironment; 3) histologically resemble human GBM and recapitulate cellular intratumoral heterogeneity; 4) have diffuse invasive growth into brain parenchyma without encapsulation; 5) be genetically similar to the original tumor, particularly preserving the expression profile of main tumor markers. In addition, the tumor model that is genetically labeled with a fluorescent or bioluminescent reporter or both would benefit from the opportunity to monitor its progression in the brain non-invasively using optical imaging techniques.

A number of different approaches have been utilized for establishing GBM models in animals, which include syngeneic rodent models, genetically engineered mouse models, human cell-line xenografts, and PDXs (3, 4). Among these models, PDXs are the most “close-to-patient” by definition and represent an important preclinical system. They demonstrate a faithful recapitulation of human GBM features such as intratumoral heterogeneity, specific molecular profile, diffuse invasion into the brain, endovascular proliferation, and pseudopalisading necrosis (5–8). While a plethora of protocols exists in the literature for the establishment of a PDX, they are not always successful, and the engraftment success rate often depends on the experience of the lab, varying from 10% to 60% on average (4, 8–11). A typical protocol includes the obtaining of single-cell suspension from the tumor, maintaining the cells in a culture for a short time (1–2 weeks), intracranial implantation into immunodeficient mice, and passaging in mice to have a stable growth. Major challenges of the PDXs are the limited availability of fresh patient tumor samples, low tumor take rate of fresh tissue or cells, difficulties of preserving viability of isolated tumor cells, propagation of cells in serum-free conditions, and long period of tumor development (several months) (4–8). Another limitation of PDXs is that they are difficult to transduce with genetic material, which can be necessary, for example, to label the tumor with fluorescent protein or luciferase (12–15).

Still, several studies demonstrate that it is possible to manipulate primary GBM cells by stably expressing the luciferase (12–14) or fluorescent protein (15) without overtly affecting their particular stem-like properties. For this, a primary cell culture is established from a patient’s GBM, transduced with the desired reporter gene and inoculated intracranially into mice. Such bioluminescently or fluorescently labeled PDX models have a high biological and clinical significance because, on the one hand, they preserve the genomic, cellular, and histopathological characteristics of the original tumors, and on the other hand, they enable a highly sensitive, long-term detection of the tumor *in vivo*. The existing examples of genetically labeled PDXs contain typically only one reporter, either luciferase or in fluorescent protein. There is only one study where the primary culture was dual-labeled with luciferase and green fluorescent protein (GFP) (14); however, GFP was used to identify tumor cells in tissue slices by fluorescent microscopy and not for *in vivo* imaging. Given that bioluminescence and fluorescence modalities have their own limitations, the development of a dual-labeled glioma PDX-expressing luciferase and a red protein is important to extend the choice of methods for *in vivo* imaging.

In recent years, there has been increasing interest in optical diagnostic methods, especially in label-free techniques, for intraoperative delineation of glioma margins (16). Since the extent of resection strongly correlates with patients’ survival, an accurate identification of tumor tissue during surgery is critically important, which, however, is difficult due to the infiltrative growth of the tumor. Macroscopic fluorescence lifetime imaging (macro-FLIM) and cross-polarization optical coherence tomography (CP OCT) are the new optical imaging methods with a high potential to differentiate between tumor and normal tissues using their intrinsic biochemical or structural features (17–19). FLIM demonstrates a high sensitivity to tissue autofluorescence and allows to differentiate endogenous fluorophores and their states based on fluorescence lifetimes (20). In terms of tumor detection, autofluorescence of the metabolic cofactors reducing nicotinamide adenine dinucleotide (phosphate) (NAD(P)H) and oxidized flavins (flavin adenine dinucleotide/flavin mononucleotide) is considered promising (21–23). Tumor cells with a higher glycolytic rate are characterized by a higher amount of NAD(P)H that is unbound with proteins and has a shorter fluorescence lifetime. Macro-FLIM has only recently been approbated for differentiation between tumorous and non-tumorous tissues. It has been found that glioma and normal brain tissue from the rat models and humans exhibit different fluorescence lifetimes in the spectral band of NAD(P)H (24). Clearly, further development of FLIM as a clinical diagnostics technique requires its validation of other, more patient-specific tumors. CPOCT is based on the detection of the polarization properties of the tissues in addition to their conventional light-scattering properties (25). The visual assessment of CP OCT images is rather difficult; therefore, the determination of optical coefficients from CP OCT data is required for the clinical application of the method (19, 26). The ability of CP OCT to differentiate tumorous tissue from the white matter has been demonstrated in several works on animal models and patients’ material (27–30). Meanwhile, development of the methods of the quantitative analysis for improving the contrast of CP OCT images remains urgent.

The present study was focused at the development of a new orthotopic human GBM model based on patient-derived GBM cells that stably co-express luciferase and a far-red fluorescent protein. We show the similarity of the established model to the original patient’s tumors in terms of histological and immunohistochemical features and demonstrate the possibility of dual non-invasive bioluminescence and fluorescence imaging of the GBM model in immunodeficient nude mice. The morphological and behavioral properties of the new model were compared with commonly used human GBM model U87 MG, which is one of the most widely studied gliomas used in experimental neuro-oncology (5–12). Furthermore, the dual-labeled PDX model was validated using advanced optical diagnostics methods macro-FLIM and CP OCT.

## Materials and Methods

### Isolation of Primary GBM Cells From Human Tumor Specimen

The primary GBM cell line has been established in the Federal Research and Clinical Center, Federal Medical and Biological Agency (Moscow, Russia). Tumor specimen has been obtained from the patient (42 years, woman) during the tumor resection. An informed written consent was obtained from the patient prior to the enrollment. This study was approved by the local human research ethics committee of the Federal Research Clinical Center of the FMBA of Russia (protocol #16 from September 30, 2017) and was performed in accordance with current legislation and the ethical standards laid down in the 1964 Declaration of Helsinki and its later amendments (31). Diagnosis was performed according to the fourth edition of the World Health Organization (WHO) classification of tumors of the central nervous system (32). Additional immunohistochemistry (IHC) staining allowed diagnosing GBM with a primitive neuronal component (**Supplementary Data; Figure S1**).

The sample of the GBM was homogenized with a surgical blade and incubated with Liberase TL (Roche, Mannheim, Germany) (50 µg/ml) for 5–10 min at 37°C in a CO_2_ incubator. After incubation, cell suspension was washed with DMEM/F12 with 2% antibiotic–antimycotic (Gibco, Grand Island, NY, USA) at 250g for 5 min. The resulting cell suspension was passed through a 100-µm cell strainer, and erythrocytes were lysed for 5 min with erythrocyte lysis buffer (ACK) (Buffer EL, Qiagen, Hilden, Germany). After that, cells were washed with DMEM/F12 at 250g for 5 min and seeded in six-well plates or 25-cm^2^ culture flasks.

### Cell Culturing

Primary GBM7 cells and U87 MG cells were cultured in RPMI-1640 medium with L-glutamine (Roswell Park Memorial Institute 1640 Medium) with addition of 10% fetal bovine serum (FBS) and 1% antibiotic–antimycotic (Gibco) on 25-cm^2^ culture flasks in a CO_2_ incubator at 37°C, 5% CO_2_, and 85% humidity. Sub-cultivation was performed twice a week by adding 1 ml of trypsin–EDTA (25%) to the plate for 2–5 min. On the third passage, GBM7 cells were characterized by immunofluorescence (IF) staining, and then lentiviral transduction was performed.

### IF Staining of Primary Human GBM Cells

For characterization of primary GBM7 cell culture, the expression of major GBM and cancer stem cell (CSC) markers was assessed. For indirect IF labeling, primary GBM cells were grown in 30-mm Petri dishes to achieve a monolayer. Then, the cells were washed with the phosphate-buffered saline (PBS), fixed with 4% paraformaldehyde for 10 min, washed, and blocked with 5% goat serum for 30 min. Then, the cell monolayer was incubated with primary antibodies at +37°C for 1 h (dilutions according to the manufacturer’s protocol, Abcam, Cambridge, MA, USA). The following primary antibodies were used: anti-glial fibrillary acidic protein (GFAP) polyclonal antibody (pAb), anti-connexin43 (Cx43) monoclonal antibody (mAb), anti-nestin mAb, anti-CD133 pAb, anti-cyclooxygenase2 (Cox2) mAb, and anti-Ki67 pAb (Abcam, Cambridge, UK). Next, the cell monolayer was washed with PBS and stained by a solution (1:500) of secondary anti-species antibodies: goat anti-mouse Alexa Fluor 488 and goat anti-rabbit Alexa Fluor 633, 595, or 536 (Abcam, UK) at 37°C for 1 h. After three washes with PBS, cell nuclei were stained with DAPI at a dilution of 1:500 for 5 min, then the cells were embedded in 50% buffered glycerol. Protein expression in primary GBM7 cells was assessed using a confocal microscope (Nikon, Tokyo, Japan).

### Lentiviral Transduction of Primary Human GBM Cells

Two reporters, Luc2 and mKate2, were selected for dual-cell labeling. For this purpose, a lentiviral vector pCDH (System Biosciences, Palo Alto, CA, USA) was used as a backbone. To obtain pCDH-Luc2-IRES-mKate2, Luc2 and mKate2-encoding sequences were linked together *via* an IRES element and placed downstream of the constitutively active human EF1a promoter (**Supplementary Figure 2**). VSV-G-pseudotyped lentiviral particles were then obtained as described in (33) and used to transduce primary human GBM cell culture at different MOIs (multiplicities of infection). Seven days after transduction, mKate2-expressing cells were bulk-sorted to obtain at least 2 × 10^5^ cells/plate to ensure the maintenance of cellular heterogeneity of the original tumor. The obtained GBM7-Luc2-mKate2 cell culture was frozen at -150°C in RPMI-1640 medium with 20% of FBS and 7% of dimethyl sulfoxide (DMSO) until the further experiments were performed.

### Phase Contrast and Fluorescence Microscopy of the Human GBM Cell Cultures

Phase-contrast and fluorescence microscopy of the GBM7, GBM7-Luc2-mKate2, and U87 MG cells was performed by Invitrogen EVOS M7000 Imaging System (Thermo Fisher Scientific, Waltham, MA, USA) at ×20 magnification on the second to third passage (10–14 days after thawing). Fluorescence microscopy images of the mKate2 signal were obtained using a Texas Red LED light cube, wavelength excitation 585/29 nm, wavelength emission 628/32nm.

### Doubling-Time Assay

To evaluate the growth rate of GBM7-Luc2-mKate2 and non-modified GBM7 cell cultures, the cells were seeded in six-well plates at the concentration of 2 × 10^5^ cells per well and after 48 h were collected and counted. The doubling time (DT) of the cells was calculated using Equation 1, where D is the duration of culturing, in hours; C1 and C 2 are the initial and final concentrations of cells, respectively (34).


(1) DT=D×ln 2/ln(C1/C2) 

### Nude Mice

The experiments were carried out on 23 athymic nude mice, female, 8-week-old, purchased from the SPF vivarium of the Institute of Biology and Biomedicine of Lobachevsky State University of Nizhny Novgorod (Russia). All animal experiments were approved by the Ethics Committee of the Privolzhsky Research Medical University (Nizhny Novgorod, Russia), approval #6 from April 17, 2019.

### Intracranial Inoculation of Dual-Labeled Human GBM Cells

GBM7-Luc2-mKate2 cells on the second to third passages (10–14 days after thawing) were implanted into the brain of nude mice by the stereotaxic system (RWD Life Science, Shenzhen, China). Mice were anesthetized with Zoletil (40 mg/kg, 50 μl, Virbac SA, Carros, France) and 2% Xyla (10 mg/kg, 10 μl, Interchemie, Venray, Netherlands). A small opening in the skull was manually drilled by using a 21-G needle (SFM Hospital Products GmbH, Berlin, Germany). GBM7-Luc2-mKate2 cells were transplanted into anatomically matched locations in mouse brains (white matter) to simulate the microenvironment of the original tumors. The coordinates of the injection were 2 mm to the right of the midline and 3 mm anterior to the lambdoidal suture (right cerebral hemisphere) and 1.5 mm deep. The technique of GBM7-Luc2-mKate2 cell inoculation, including cell dose per mouse and parameters of stereotaxic injection, has been optimized by the authors and is described in detail in the Results section. After injection, skin was closed with suture Ethibond Excel 6/0 (Ethicon (Johnson Johnson), New Brunswick, NJ, USA).

U87 MG cells (5 × 10^5^ cells per mouse resuspended in 10 μl PBS, at the rate of 2 μl/min) were implanted the same way and using the same coordinates of the mouse brain.

### *In Vivo* Bioluminescence and Fluorescence Imaging

Monitoring of the growth of GBM7-Luc2-mKate2 xenografts in mice was performed by *in vivo* bioluminescence and fluorescence imaging using an IVIS Spectrum imaging system (Caliper Life Sciences, Hopkinton, MA, USA). *In vivo* macroscopic images were acquired two to three times per week starting from the fourth day after tumor cell inoculation. During *in vivo* imaging, the mice were anaesthetized with 2.5% isoflurane in oxygen. The bioluminescence signal was detected 15 min after intraperitoneal (IP) injection of D-luciferin, Sodium Salt (BioVision, Milpitas, CA, USA), 150 mg/kg at exposure time from 2 to 5 s. Fluorescence of mKate2 was excited at the wavelength of 570/30 nm and detected at 640/20 nm at exposure time of 2 s. Before fluorescence imaging of mKate2, the scalp was surgically opened. The bioluminescence and fluorescence images were analyzed using Living Image 4.2 software (Caliper Life Sciences, USA). The bioluminescence signal was quantified by using total flux (photons/s) in a region of interest (ROI), manually drawn to outline the whole area of the signal from the brain tumor. For quantification of the fluorescence signal of mKate2 from the tumor, the brightest area around the place of GBM cell injection was selected as an ROI and the average radiant efficiency ((photons/s/cm²/sr)/(μW/cm²)) was calculated and normalized to its corresponding value measured before the injection.

### Intravital Confocal Microscopy

During the procedure, the mice were anaesthetized with 2.5% isoflurane in oxygen. For intravital microscopy, the animals were placed in a stereotaxic frame (Stoelting, Wood Dale, IL, USA), and soft tissues were dissected in the projection of the skull bones. A hole of 5 mm in diameter was drilled in the center of the parietal bone above the inoculated glioblastoma and covered with a cover glass (thickness is 0.15 mm). A catheter for infusion of fluorescein isothiocyanate (FITC), which served as a contrast agent for microvessels, was inserted into the tail vein of the mice. Then the animals were immediately investigated using an A1 MP+ multiphoton confocal microscope (Nikon, Japan). To visualize peripheral blood granulocytes, fluorescently labeled antibodies to lymphocyte antigen 6 complex locus G6D (Ly6G) were also injected *via* the catheter. To visualize tumor cells and tumor microvessels located in the cortex, several tumor fragments were scanned for 120 min from the moment of injection (1 frame 512 × 512 per 40 s). Further, all the obtained images were stacked to obtain a time-lapse 3D model of the tissue. To improve the quality of *in vivo* images, the obtained z-stacks were processed in NIS Elements software (Nikon, Japan).

### FLIM on the Macroscale of the *Ex Vivo* Mouse Brain

A confocal macro-FLIM system, described earlier in elsewhere (17, 18, 24), was utilized to obtain autofluorescence images of *ex vivo* mouse brains with GBM7-Luc2-mKate2. The brain samples from U87 MG-bearing mice and intact mice without a tumor were also investigated. The mice were euthanized with 90% isoflurane on the 14th–15th days of tumor growth. The brains were gently removed and divided in two tissue blocks in frontal plane using a scalpel. The brain tissue was analyzed by FLIM immediately.

The tissue autofluorescence was excited by a picosecond diode laser (BDL-375-SMN, Becker & Hickl GmbH, Germany) at the wavelength of 375 nm with the power incident on a sample of 18 µW. The wavelength of excitation was selected based on the absorption of NAD(P)H at this wavelength. The signal was registered in the spectral range determined by a bandpass filter 460/50 nm (Chroma, Foothill Ranch, CA, USA). Image acquisition time was 120 s, which allowed to collect >5,000 photons per decay curve without binning.

Fluorescence images of red fluorescent protein mKate2 were obtained for brain samples with GBM7-Luc2-mKate2 to identify the tumor zone on the FLIM images. The signal of mKate2 was excited by a picosecond diode laser (BDS-594-SM, Becker & Hickl GmbH, Germany) at 594 nm with the power incident on a sample of 5 µW and detected at 610 nm, determined by a long pass filter (610 LP, Chroma, USA). Freshly excised brains from the intact mice were used as a control.

SPCImage software (Becker & Hickl GmbH, Germany) was used to process the FLIM data. The fluorescence decay curves were fitted with a bi-exponential decay model providing short- and a long-lifetime components (τ_1_ and τ_2_, respectively), and the relative amplitudes of the lifetime components (a_1_ and a_2_, where a_1_ + a_2_ = 100%). The amplitude-weighted mean fluorescence lifetime was also calculated as shown in Equation 2.


(2) $$\tau_{m}=a_{1}\times \tau_{1}+a_{2}\times \tau_{2}$$

For all data presented here, the quality of the fit, the χ^2^ value, was within the appropriate range of 0.8–1.2. Tumor areas and areas of cortex and the white matter were selected as ROIs in each image of the mouse brain.

### CP OCT

A spectral domain CP OCT device (Institute of Applied Physics of the Russian Academy of Sciences, Nizhny Novgorod, Russia) with cross-polarization detection was used in the study (25, 35). The central wavelength of the light source used for OCT is 1,310 nm with an average power of 20 mW. The axial and lateral resolution in air is 10 µm and 15 µm, respectively. The probing beam is circularly polarized. The device has a scanning rate of 20,000 А-scans/s and performs 2D lateral scanning within an area of 2.4 × 2.4 mm^2^ to obtain the 3D distribution of backscattered light in the polarization with the same and reversed rotations of the electric-field vector (25).

CP OCT images of the brain sections with GBM7-Luc2-mKate2 were acquired *ex vivo* immediately after macro-FLIM. Tissue blocks were oriented in such a way that its upper surface corresponded to the cut in the frontal plane. Scanning was performed in contactless mode. 3D images in co-polarization and structural 2D (B-scans and en-face) images in co- and cross-polarizations are displayed on the personal computer monitor in the process of scanning. A sequential scanning of the entire sample surface was performed row by row with overlapping images. Then, individual *en-face* color-coded maps based on optical coefficients calculation from each A-scan were constructed. Two optical coefficients—attenuation in co-channel (Att_co-_) and in cross-channel (Att_cross-_)—were calculated according to the method described in (36). The optical coefficients were calculated in the same predefined depth range starting from ~70 µm below the tissue surface (pixel #10) to a depth of ~350 µm below the tissue surface (pixel #50). For better identification of the tumor localization, *en-face* color-coded maps obtained from one sample were reconstructed into a single, complete picture of the mouse brain.

Color-coded maps provide information about the tissue properties in the range of specified depths; therefore, they are easier to interpret and show the tumor more contrast than the original intensity OCT images, for the analysis of which only one plane from the tissue surface is selected. Intensity OCT images are not presented in this paper.

### Histological Analysis and Immunohistochemistry

After imaging procedures, brain sections with GBM7-Luc2-mKate2 and U87 MG were fixed in formalin for 24 h, embedded in paraffin following a standard protocol, and sectioned parallel to the optical plane. For the evaluation of tissue histology, 7-μm-thick paraffin sections were routinely stained with hematoxylin and eosin (H&E).

For IHC, 4-μm paraffin sections were used. IHC staining was performed automatically on the Bond-Max immunohistotainer (Leica Biosystems, Newcastle, UK) using the Bond Polymer Refine Detection imaging system (protocol F). The staining protocol included preliminary deparaffinization of the sections and unmasking for 20 min at a temperature of 98°C–99°C. Next, the sections were incubated with primary antibodies for 15 min. Monoclonal antibodies to Ki67 clon MIB1 (Dako, Glostrup, Denmark), to p53 clon DO7 (Leica Biosystems, UK), and to GFAP clon GA5 (Leica Biosystems, UK) were studied.

All tissue slides were examined with a Leica DM2500 microscope (Leica, Japan) at a ×10 and ×20 magnifications.

### Statistical Analysis

The mean values (M) and standard error of the mean (SEM) were calculated. Differences between groups were analyzed using the Mann–Whitney U test in GraphPad Prism 9.00 (GraphPad, San Diego, CA, USA) software. A p value ≤0.05 was considered statistically significant.

## Results

**Figure 1:**
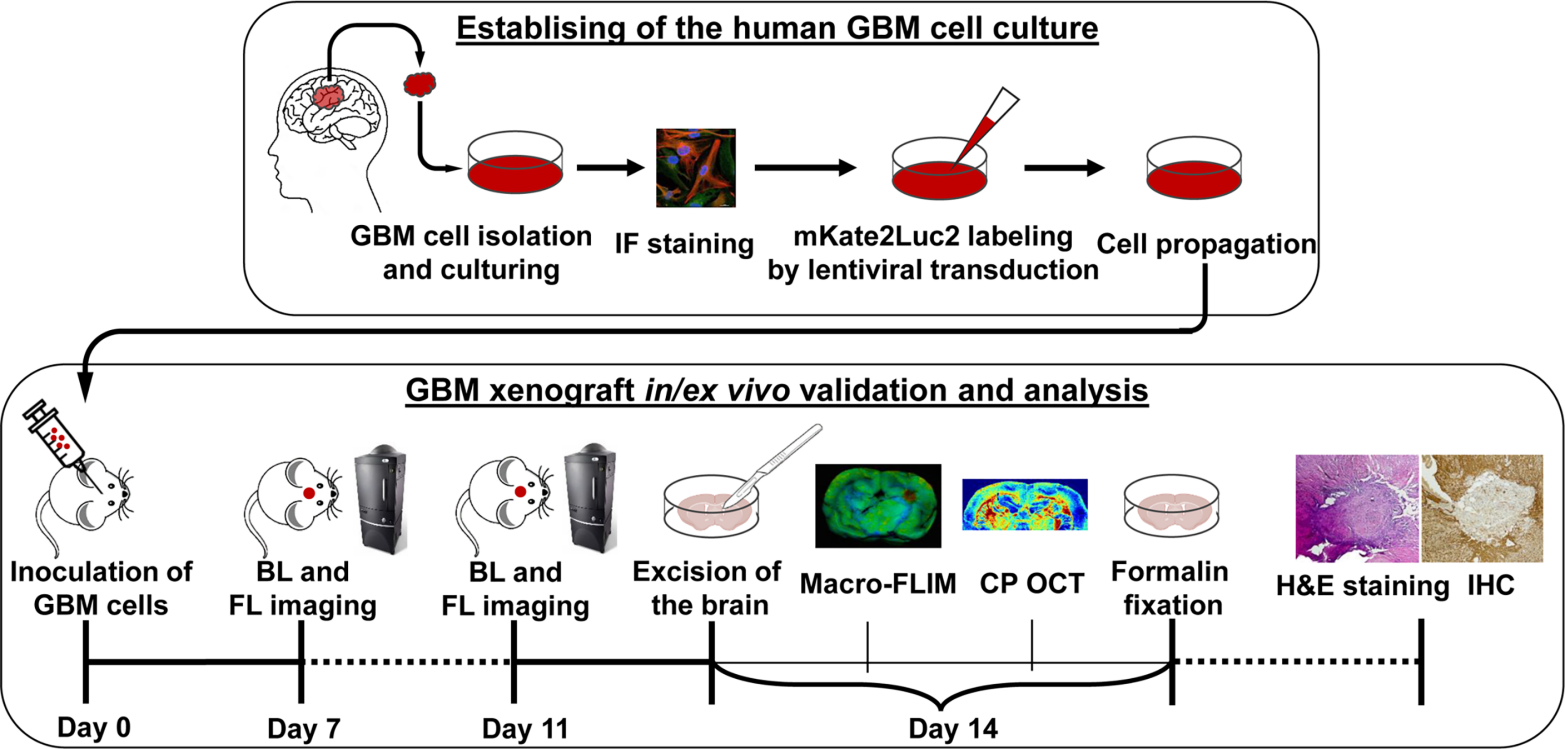
Design of the experiment. Establishing of the cell culture from the human tissue sample of GMB included isolation from the freshly resected brain tumor specimen, immunofluorescence (IF) staining, lentiviral transduction for dual labeling, cell culturing, and propagation. Validation and analysis of GBM7-Luc2-mKate2 xenograft included *in vivo* bioluminescence (BL) and fluorescence (FL) imaging, *ex vivo* macro-FLIM, CP OCT, histological evaluation, and immunohistochemistry (IHC).

The experimental design is demonstrated in **Figure 1**. At the first stage, cell culture was obtained from the freshly resected brain tumor specimen and underwent lentiviral transduction for dual labeling. The second stage included GBM7-Luc2-mKate2 cell inoculation into the mouse brain with further *in vivo* monitoring of tumor growth, *ex vivo* macro-FLIM and CP OCT imaging, and histological evaluation.

### Characterization of the Human GBM7 Cell Culture

**Figure 2:**
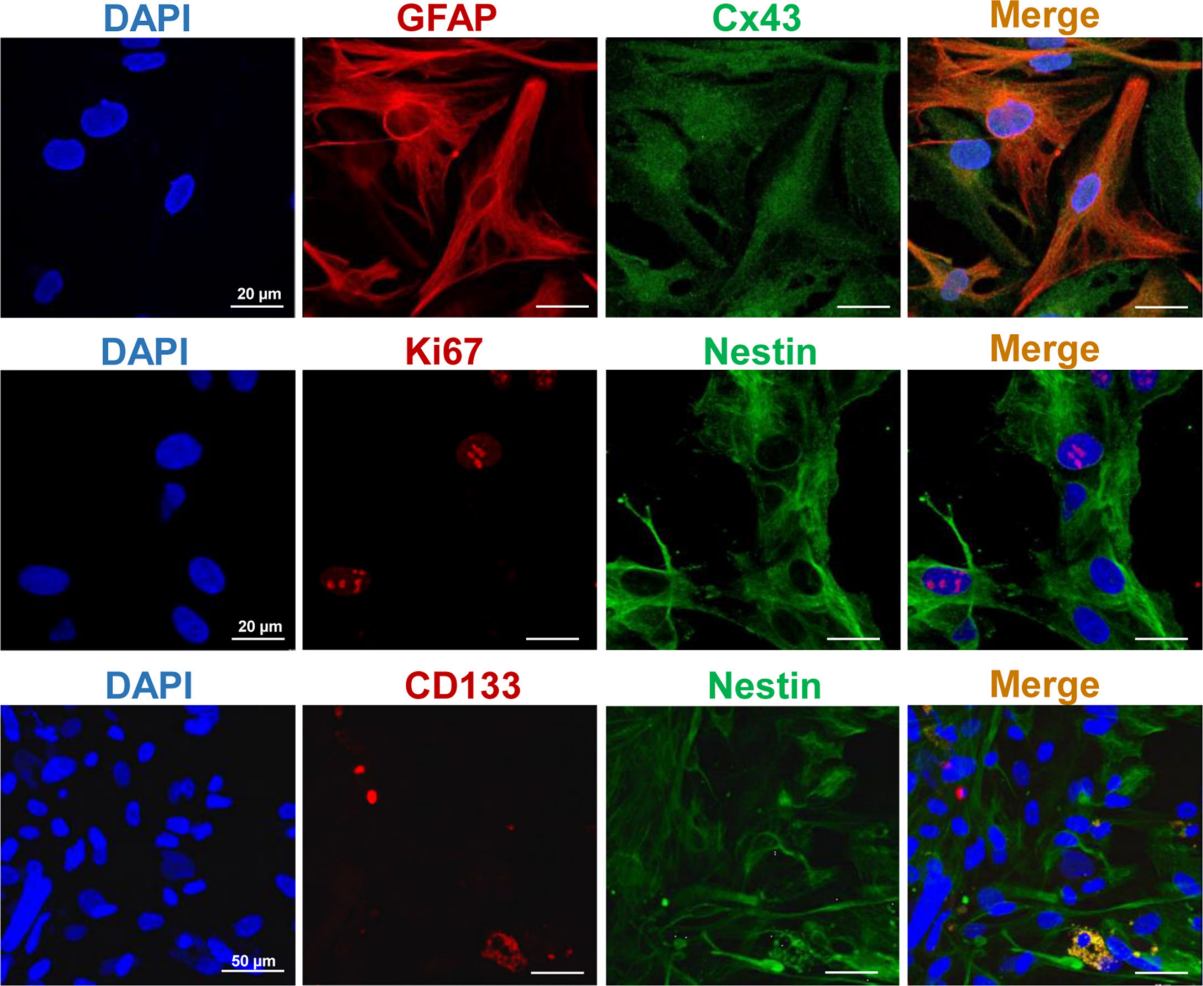
Representative IF staining images of the primary human GBM cells. Cell nuclei were counterstained with DAPI. Bars are applicable to all images in the row.

To characterize the immunophenotype of the primary human GBM7 cell culture, the expression of major glial and CSC markers was examined with IF staining (**Figure 2**). The cell culture demonstrated a moderate expression of GFAP, the most widely used marker of astroglial cells, and Cx43, a gap junction protein, associated with high invasive potential (37, 38). Concerning CSC markers, strong expression of nestin, an intermediate filament protein produced in stem/progenitor cells in the mammalian CNS during development and re-expressed in the adult organism under certain pathological conditions including neoplastic transformation, was detected. Furthermore, a small amount of CD133+ cells was observed. The proliferation index estimated with Ki67 staining was more than 5% (7%–10%).

**Figure 3:**
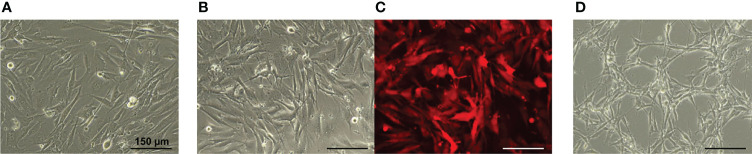
Phase contrast **(A, B, D)** and fluorescence microscopy **(C)** of the GBM cell monolayer. **(A)** GBM7 cells. **(B, C)** GBM7-Luc2-mKate2 cells. **(D)** U87 MG cells. Bars are applicable to all images in the row.

Concerning the cell morphology, the primary human GBM7 cell monolayer displayed diffuse cell distribution and moderate cell polymorphism (**Figure 3A**). Numerous large cells with a fusiform morphology (fibroblastic-like cells) were found. Furthermore, triangular, oval, round, or irregularly shaped cells were also observed. All cells presented numerous and extensive cytoplasmic prolongations and pronounced cytoplasmic granularity.

The lentiviral transduction of human GBM7 cells did not affect the cell morphology and the growth rate. Similarities in cell morphology between the patient and dual-labeled cells are clearly seen (**Figure 3B**). GBM7-Luc2-mKate2 cell culture preserved the diffuse distribution in monolayer, shape, and cytoplasm features of the primary cells. The strong fluorescence signal of mKate2 was detected. There were no statistically significant differences between the DT of modified and non-modified GBM7 cells (31.9 ± 0.9 h versus 32.8 ± 0.7 h correspondingly).

In contrast, the morphology of the U87 MG cells and their arrangement in space are rather different (**Figure 3C**). The U87 MG cell line demonstrated a more ordered cell distribution in monolayer and less cellular heterogeneity. Elongated cells with a fusiform morphology formed a honeycomb-like structure with clusters. The cytoplasm was less granular and presented extensive prolongations.

### Establishment of Intracranial Dual-Labeled Human GBM in Mice

According to the standard techniques, human GBM cells are inoculated in a mouse brain in the amount of up to 5 × 10^5^ tumor cells in 2–5 μl of serum-free media (the final concentration of the cell suspension is ~1–1.6 × 10^5^ cells/μl) at the rate of 1 μl/min (10, 11). In our study, different cell doses and rates of injection were tested to generate tumors from patient-based GBM7-Luc2-mKate2 cells.

At the recommended cell dose of 5 × 10^5^ cells per mouse, the development of GBM7-Luc2-mKate2 in the mouse brain was not observed. Therefore, we increased the cell dose to 2 × 10^6^ cells per mouse. The injection volume and rate were respectively changed to 20 μl per mouse and 2 μl/min, keeping the final concentration of cell suspension of 1 × 10^5^ cells/μl, since the loading of a denser suspension into the syringe was problematic. Moreover, we resuspended tumor cells in PBS instead of serum-free medium; otherwise, unwanted air bubbles were observed to form during pipetting.

The depth of the GBM7-Luc2-mKate2 cell injection was selected as 1.5 mm for *in vivo* tumor visualization by fluorescence imaging. It was found that, at a deeper localization (around 2.5 mm) we could not detect the tumor development by fluorescence (**Supplementary Figure S3** **, mouse#2 and #3**).

Therefore, the optimized technique for intracranial inoculation of GBM7-Luc2-mKate2 included the injection of 2 × 10^6^ tumor cells per mouse, resuspended in 20 μl of PBS, at the rate of 2 μl/min at a depth of 1.5 mm. This protocol allowed the formation of GBM in the nude mouse brain in 96% cases (22 out of 23 mice).

### *In Vivo* Monitoring of Tumor Development by Bioluminescence and Fluorescence Imaging

Dual labeling with Luc2 and mKate2 provided the opportunity to monitor the development of the tumor in live mice non-invasively and verify the presence of the tumor in the brain samples *ex vivo*. Bioluminescence imaging, by labeling xenografts with luciferases, is an accurate and powerful technique to assess intracranial GBM non-invasively, at the whole-body level. On the other side, fluorescence imaging with fluorescent proteins does not require additional substrates and employs more common systems, including fluorescence microscopy. Therefore, the dual-labeled tumors combine the advantages of both approaches and provide more experimental flexibility.

**Figure 4:**
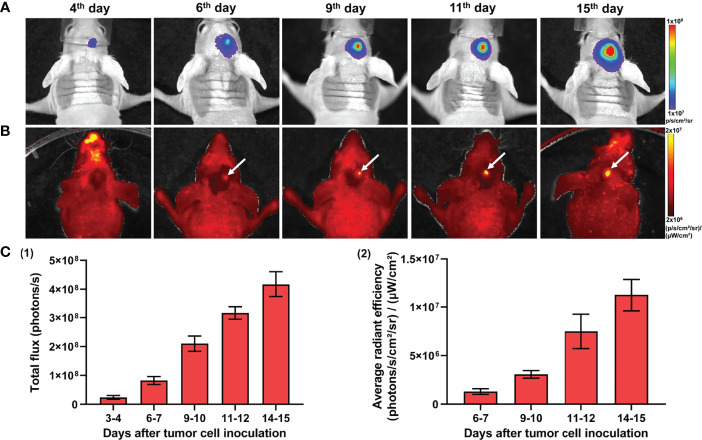
*In vivo* bioluminescence and fluorescence imaging of GBM7-Luc2-mKate2 xenografts. Representative bioluminescence **(A)** and fluorescence **(B)** images of the tumor-bearing mice from the 4th to 15th days after tumor cell inoculation. **(C)** Quantification of the bioluminescence (1) and fluorescence (2) signal during the tumor development. Mean ± SEM, n = 5–7 tumors.

*In vivo* monitoring of GBM7-Luc2-mKate2 xenograft growth was performed by both techniques, fluorescence and bioluminescence imaging. Bioluminescence imaging allowed the visualization of tumors in the mouse brain non-invasively, starting from the 4th day after tumor cell inoculation. As tumors grew, the increase in bioluminescence signal was detected (**Figures 4A, C** **)**. The alternative way to detect a tumor was fluorescence imaging. To visualize the tumor by fluorescence, the skin flap over the tumor was surgically opened. Using fluorescence mode, tumors could be detected from day 7. **Figure 4** demonstrates representative bioluminescence and fluorescence images of a mouse with GBM during tumor development and the increase in corresponding signals from the tumors.

The expression of a far red mKate2 fluorescent protein allows GBM7-Luc2-mKate2 xenografts to be visualized using intravital confocal microscopy. For this purpose, parietal bone fragments were removed in three mice 7 days after glioblastoma inoculation. Subsequent intravital microscopy allowed visualization of intracranial tumor cells and peripheral vessels after intravenous injection of FITC and Ly6G-positive mononuclear cells (**Supplementary Figure S3**). Thus, our model is applicable to study the migration of immune cells into the tumor in real time.

### Histopathology and IHC Analysis of Human GBM Xenografts

**Figure 5:**
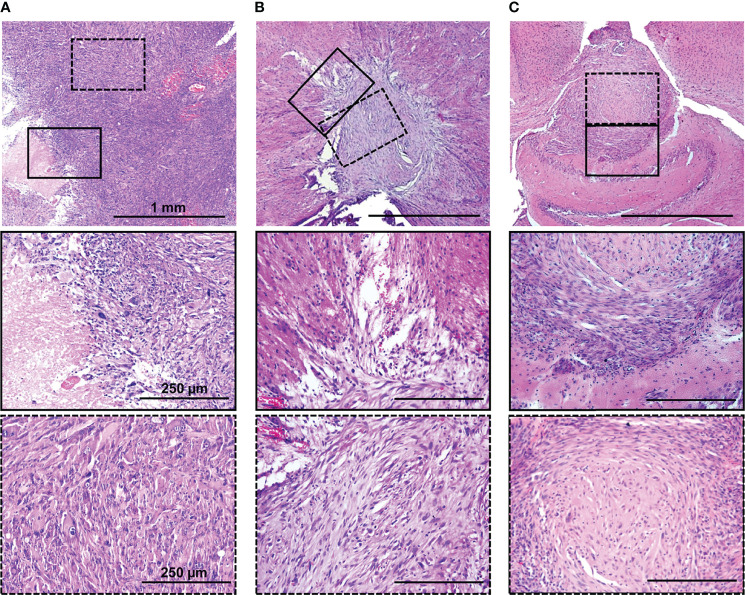
H&E staining of original patient’s tumor **(A),** dual-labeled human GBM **(B)**, and U87 MG **(C)** xenografts. Enlarged regions are indicated by the black squares on the lower-magnification panel. Bars are applicable to all images in the row.

The histopathology of the GBM xenografts obtained was examined and compared with that of the original patient’s tumor and the standard U87 MG model. A histopathology analysis of the mouse brains xenotransplanted with dual-labeled human GBM cells showed the location of the tumors 1.5–1.7 mm deep, with prominent invasion into the white matter tracts and cortex and unclear boundaries (**Figure 5B**; **Supplementary Figure S4**). The presence of regions of dystrophically altered brain parenchyma among the tumor tissue was shown. On the tumor periphery, several full-blooded sinusoids and hemorrhages were detected. At the cellular level, dual-labeled human GBM xenografts displayed pronounced cellular and nuclear heterogeneity which was remarkably similar to the original patient’s GBM (**Figure 5A**). The tumors were composed of the cells of various sizes and shapes. Large elongated cells with oval light nuclei and a large number of small cells with rounded hyperchromic nuclei were found. Giant cells with one large nucleus or several hyperchromic nuclei were also observed. The nuclei were round, oval, bean-shaped, or shapeless; hyperchromic; or with moderate chromatin content. The cytoplasm of cells was weakly basophilic.

The patient’s tumor tissue had a dense structure with a few blood vessels, with regions of necrosis and hemorrhages. Altered swollen cells with vacuolization of the cytoplasm and destruction of the nucleus were found. Cellular and nuclear polymorphism was pronounced. The presence of giant cells with one large nucleus or several hyperchromic nuclei was detected. The tumor had high mitotic activity.

Both GBM xenografts and the patient’s tumor demonstrated a high degree of invasion into the white matter tracts and in the cortex and unclear boundaries.

In contrast, the U87 MG model had a more homogeneous compact structure with clear borders. The tumors consisted of cells with a small amount of oxyphilic cytoplasm, with large oval or round light nuclei with finely dispersed chromatin and nucleoli. The central region had a less dense structure and dystrophically changed cells. Tumor vascularization is moderately expressed, represented by homogeneously distributed small full-blooded vessels. Numerous mitoses among proliferating cells were observed.

**Figure 6:**
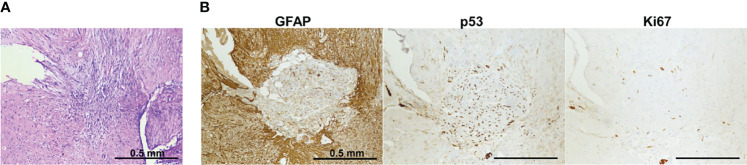
IHC characterization of GBM7-Luc2-mKate2 xenograft. Representative H&E **(A)** and IHC **(B)** stained sections of a whole tumor. Bars are applicable to all images in the row.

To evaluate whether dual-labeled human GBM xenografts maintained the immunophenotype of the original tumor, the expression of glial and tumor markers was examined with IHC staining. The xenografts were characterized by the presence of GFAP, the high expression of p53 in a large proportion of cells (60%–70%), and a moderate proliferation index (Ki-67+, 20%–22%) (**Figure 6**). A moderate diffuse expression of GFAP and a strong diffuse expression of p53 in GBM xenografts closely resembled the distribution and staining intensities of these markers in the original patient GBM with a primitive neuronal component, whereas the Ki67 score in xenografts was lower than in the patient’s tumor (20-22% versus 40%–45%) (**Supplementary Data**).

Therefore, the dual-labeled GBM xenografts were generally similar to the original patient’s GBM in their histopathological and immunohistochemical characteristics (**Table 1**).

**Table 1:** The summarized histopathological and IHC characterization of GBM7-Luc2-mKate2 xenograft compared to the original tumor.

	Patient tumor	Dual-labeled human GBM xenograft
**H&E staining results**		
Tumor invasion	++	++
Cellular heterogeneity	++	++
Vascularization	++	+
Mitotic activity	++	+/-
**IHC results**		
GFAP	+	+
p53	++	++
Ki67	40%–45%	20%–22%

### Macro-FLIM of Human GBM Xenografts

Using the GBM7-Luc2-mKate2 xenografts, we tested macro-FLIM as a novel diagnostics method based on the endogenous contrast. Fluorescence was detected from freshly excised mouse brains in two spectral channels, corresponding to NAD(P)H (ex. 375 nm, reg 435–485 nm) and mKate2 (ex. 594 nm, reg 610 nm). Fluorescence of mKate2 was used to accurately identify the tumor. In a search for differences between GBM and normal brain tissue, we compared the parameters of autofluorescence lifetime measurements in the tumor zone with the tumor-distant cortex and the white matter (**Supplementary Table S1**).

**Figure 7:**
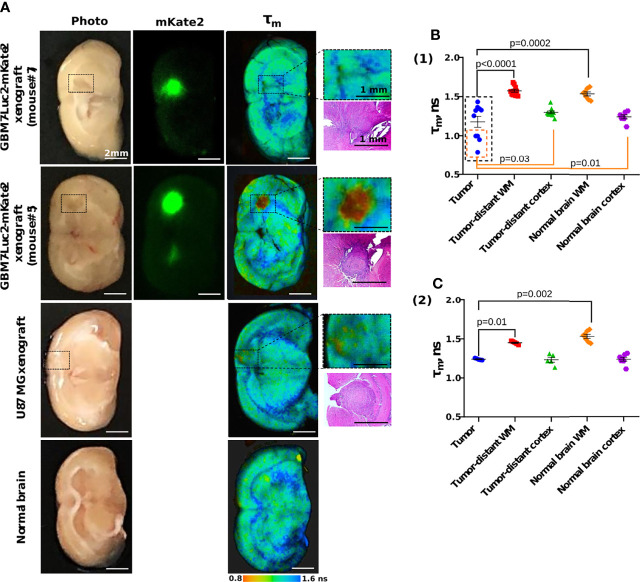
Macro-FLIM of human GBM xenografts and normal brain. **(A)** Representative auto fluorescence time-resolved images of GBM7-Luc2-mKate2 xenografts, U87 MG xenograft, and normal mouse brain without tumor. Enlarged regions with a tumor are indicated by the black squares on the lower-magnification panel. The corresponding H&E-stained section is presented under each enlarged region. **(B)** Quantification of the mean fluorescence lifetime tm in the NAD(P)H spectral channel in (1) dual-labeled human GBM xenografts and (2) U87 MG xenografts and normal brain. Scatter dot plot displays the measurements for individual animals (dots) and the mean and SEM (horizontal lines). WM is a white matter.

All the fluorescence lifetime parameters (τ_m_, τ_1_, τ_2_, a_1_, a_2_) of the tumors demonstrated differences from the white matter in all mice (**Table 2**). GBMs had shorter lifetime values τ_1_ and τ_2_ and greater contribution of short lifetime component a1 compared to the tumor-distant white matter and white matter of normal brains, resulting in shorter mean lifetime tm (tm = 1.18 ± 0.07 ns versus 1.57 ± 0.02 ns and 1.54 ± 0.03 ns, p < 0.0001 and p = 0.001 correspondingly) (**Figure 7B**).

**Table 2:** Autofluorescence lifetimes in GBM, white matter, and cortex.

Mean ± SEM	*τ_m_*, ns	*τ_1_*, ns	*τ_2_*, ns	*a_1_*, %	*a_2_*, %
GBM7-Luc2-mKate2 **xenograft**					
**Tumor** **(all mice)**	1.18 ± 0.07 *p < 0.0001 p = 0.0002	0.52 ± 0.03 *p < 0.0001 p = 0.002	3.2 ± 0.1 *p = 0.001	75.4 ± 1.2 *p = 0.003 p = 0.04	24.6 ± 1.2 *p = 0.003 p = 0.04
**Tumor-distant white matter** **(all mice)**	1.57 ± 0.02	0.72 ± 0.01	3.78 ± 0.01	71.1 ± 0.6	28.9 ± 0.6
**Tumor-distant cortex** **(all mice)**	1.29 ± 0.01	0.64 ± 0.01	3.33 ± 0.05	75.1 ± 0.4	24.9 ± 0.4
**Tumor** **(mice #2, 3, 5, 6)**	0.93 ± 0.03 **p = 0.03 ^#^p = 0.01	0.41 ± 0.02 **p = 0.03 ^#^p = 0.01	2.92 ± 0.06 **p = 0.03	79.1 ± 0.3 **p = 0.03 ^#^p = 0.01	20.9 ± 0.3 **p = 0.03 ^#^p = 0.01
**Tumor-distant cortex** **(mice #2, 3, 5, 6)**	1.289 ± 0.004	0.646 ± 0.003	3.3 ± 0.03	75.4 ± 0.3	24.6 ± 0.3
**U87 MG xenograft**					
**Tumor**	1.24 ± 0.01 *p = 0.01 p = 0.002	0.599 ± 0.003 *p = 0.04 p = 0.02	3 ± 0.01 *p = 0.04 p = 0.02	72.9 ± 0.07	27.1 ± 0.07
**Tumor-distant white matter**	1.449 ± 0.009	0.745 ± 0.008	3.39 ± 0.05	73 ± 0.6	27 ± 0.6
**Tumor-distant cortex**	1.23 ± 0.03	0.67 ± 0.016	3.1 ± 0.1	76.7 ± 0.1	23.3 ± 0.1
**Normal brain without tumor**					
**White matter**	1.54 ± 0.03	0.72 ± 0.03	3.5 ± 0.2	69.6 ± 1.7	30.3 ± 1.7
**Cortex**	1.23 ± 0.03	0.67 ± 0.02	3.1 ± 0.1	76.7 ± 0.9	23.3 ± 0.9

*Statistically significant difference from the tumor-distant white matter; ** - from the tumor-distant cortex; ^#^ - from the normal brain white matter; ^##^ - from the normal brain cortex.

A comparison of fluorescence lifetimes of tumors and cortex in individual brains showed that only four out of 10 tumors (mice #2, 3, 5, 6, indicated by the orange square in **Figure 7B**) demonstrated a statistical difference from the tumor-distant cortex. GBM in those mice were characterized by shorter lifetimes τ_1_ and τ_2_ and greater a1 compared to the tumor-distant cortex and to the cortex of normal brains, resulting in shorter τ_m_ (τ_m_ = 0.93 ± 0.03 ns versus 1.289 ± 0.004 ns and 1.23 ± 0.03 ns, p = 0.004 and p = 0.0002 correspondingly).

Precise matching of the macro-FLIM images with corresponding histological slides showed no principal differences in tissue structure between the tumors that differed from the cortex and those that did not (**Supplementary Figure S4** **)**.

U87 MG tumors showed differences in fluorescence lifetime from the tumor-distant and normal white matter, but not from the cortex (**Figure 7B**).

Note that although all the GBM xenografts were generated from the same primary cell culture, their autofluorescence lifetime parameters significantly varied between tumors, in contrast to those of normal brain tissues and U87 MG xenografts.

### CP OCT

**Figure 8:**
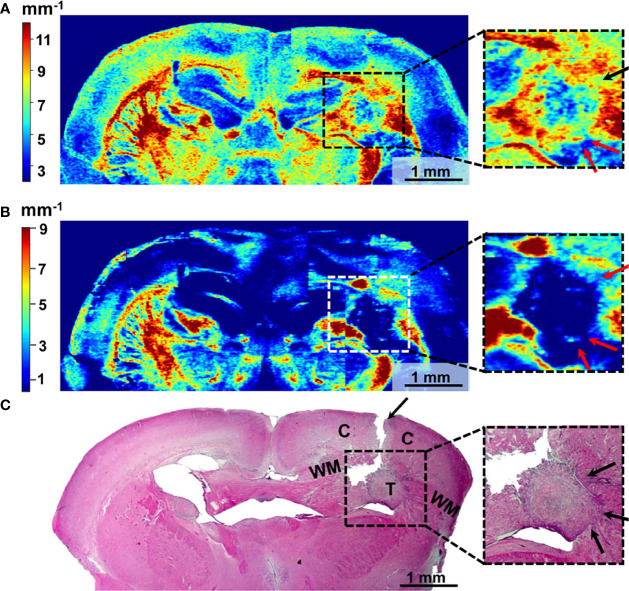
Wide-field OCT color-coded maps of the mouse brain with GBM7-Luc2-mKate2 tumor **(A, B)** and corresponding histology **(C)**. Color-coded maps based on calculation of two optical coefficients: attenuation in co-channel (Att_co-_) **(A)** and in cross-channel (Att_cross-_) **(B)**. Perifocal areas of high cancer density are marked with arrows (see enlarged fragments). T, tumor; C, cortex; WM, white matter.

The *ex vivo* samples of dual-labeled human GBM were also tested with CP OCT. Color-coded maps of optical coefficient distribution showed that areas of tumor growth can be detected among the normal white matter of the brain (**Figures 8A, B**).

The localization of the tumor on the color-coded maps (**Figures 8A, B** **)** coincides with that on the histological sections (**Figure 8C**). However, there may not be an exact match between the color-coded maps and the histological section because of the sample deformation during the histology preparation.

The tumor with a predominantly cellular component is characterized by lower values of the attenuation coefficients in co- (**Figure 8A**) and cross- (**Figure 8B**) channels compared to the white matter, having the structure of organized fibers and manifesting high scattering and polarization properties. Therefore, the tumor is well contrasted in the right hemisphere of the brain compared to the structures formed by the normal white matter in the left hemisphere. Notably, the distribution of optical coefficients reveals not only the tumor core but also tumor infiltrative growth. Areas of high cancer density indicated by arrows in histology (**Figure 8C**, enlarged fragments) have the same optical attenuation values as the tumor (**Figures 8A, B** arrows in enlarged fragments), and in cross-channel (**Figure 8B**) the differences between the cancer cells and myelinated fibers are more visible.

## Discussion

Here, we developed a new orthotopic human GBM model in immunodeficient nude mice using the patient-derived GBM cells genetically labeled with bioluminescent and fluorescent reporters for *in vivo* optical imaging. The novel GBM model, on the one hand, preserves the key features of a patient’s tumor such as the invasion growth pattern, cellular heterogeneity, and expression profile of tumor markers, and on the other hand, enables detection and following of the tumor growth *in vivo* in real time.

For a long period of time, preclinical models of human GBM were generated from stable, immortalized human cell lines developed back in the 1960s (39, 40). Although human stable cell-line xenografts are easy to work with, can be continuously maintained in a cell culture, and better mimic human GBM histopathology than mouse cell lines, they have several serious disadvantages. Specifically, similar to other cell-line models, human GBM cell lines exhibit genetic drifts and alterations in their transcriptomes, lose cellular heterogeneity, and lose the ability to invade adjacent tissues following prolonged culturing with serum (41, 42). Our results on histological analysis of human GBM U87 MG corresponded to the published data (43, 44) and showed a rather homogeneous, compact structure of the tumor without pronounced cellular heterogeneity and nuclear atypia. Tumor vascularization was moderately expressed, and no pseudopalisading necrosis was found. Nevertheless, high mitotic activity typical for human GBM was present. In addition, U87 MG tumors had clear and distinct borders without diffuse invasion, which is consistent with other studies.

In the field of preclinical glioma models, there is increasing interest in establishing xenografts from patient-derived tumor tissue (5–8). Besides providing relevant biological characteristics, PDXs have become a valuable tool for the development of new diagnostic technologies and an accurate prediction of the clinical outcome of novel therapeutic strategies (7, 45). The important property of PDXs is their ability to recapitulate the original tumor features such as heterogeneous histology, molecular profile, malignant phenotypes and genotypes, tumor vasculature, and invasive capacity. Classical PDX is established as a model at low passage numbers, passed in animals, and/or propagated in cell culture conditions (8, 10, 11). The main challenges with PDX are that the availability of fresh tumor tissue is often limited and the primary tumor cells have low viability, which reduces the reproducibility of experimental results and success rate for generating intracranial tumors (5, 6). In our work, we created a reproducible cell culture based on the patient GBM cells capable of efficiently forming tumors in nude mice in as little as 5–7 days after orthotopic implantation. Owing to the ability to grow in a culture, the new patient’s cell-based model overcomes the difficulties of “classical” PDX in terms of long period of tumor development, as well as labor-intensive and time-consuming establishment procedure.

The direct orthotopic engraftment of fresh primary tumors in immunodeficient animals has been shown to better recapitulate the cellular, molecular, and clinical phenotypes of different human cancers (6, 8). Compared to the subcutaneous location, the orthotopic location provides the appropriate microenvironment to more accurately mimic the natural tumor invasiveness and the metastatic process (46, 47). Wang and Sordat were among the first ones to describe the technique of orthotopic implantation for human colorectal tumor in nude mice in 1982 (48). Since then, orthotopic models have been established for all major tumor types including gliomas (49). For brain tumors, however, orthotopic injection into mouse brains remains a surgically challenging procedure (50–52). Along with additional obstacles such as low tumor take rate of fresh tumors and long period of tumor formation, this might have been responsible for the lack of PDX models of gliomas. We optimized the orthotopic injection technique by increasing the cell dose per mouse from the recommended 5 × 10^5^ cells to 2 × 10^6^ cells to ensure the high success rate (96%) of model development at 5–7 days after tumor cell inoculation. The new human GBM model preserved such histologic hallmarks of the original patient’s GBM as pronounced cellular polymorphism and nuclear atypia. Pseudopalisading necrosis and vascular hyperplasia typical for human GBM (4) were not observed. The possible reason is the early growth period of our GBM model (13–14 days of growth).

Deep infiltration of human GBM is mediated by repeated cycles of coordinated mass migration of cells away from a nutrient- and oxygen-impoverished microenvironment, angiogenesis, vascular collapse, and new cellular migration in search of a more hospitable microenvironment (2). This growth pattern not only obstructs with accurate surgical removal but also contributes to resistance, making treatment of these tumors difficult (53). Our model demonstrated a high invasion into the white matter tracts and in the cortex and unclear boundaries, similar to patient GBM.

It is known that prolonged cell culturing with serum results in a genetic drift and a decrease in cellular heterogeneity (41). Concerning human stable GBM cell lines (e.g., U87 MG, U251) that were initially established from a patient with GBM in the 1960s, these have been cultivated for decades and lost some of their ability to accurately recapitulate the biology of human GBM (42). With newly established cell cultures, this can be avoided by maintaining cells in serum-free neural stem cell media, allowing for the maintenance of a human GBM phenotype (3, 4). In our study, the patient GBM7-Luc2-mKate2 cells were cultured on the serum-based medium for 2 years, while preserving the key histopathological features of human GBM, such as diffuse infiltrative growth, cellular heterogeneity, and expression profile of the origin tumor. Nevertheless, to avoid losing the resemblance to the original patient GBM, it is better to further cultivate the cell line in the serum-free neural stem cell media.

The genetic modification of patient-derived glioma cells with bioluminescent and/or fluorescent reporter opens up the possibility to monitor the growth of the intracranial tumors in mice *in vivo* using optical imaging techniques (12–15). The benefit of using bioluminescence or fluorescence imaging is that the tumor development can be observed rapidly, over time without the animal being sacrificed, thus allowing stratification of mice and timely exclusion of animals without tumors (54–56). Fluorescence and bioluminescence imaging have their own advantages and disadvantages. The advantage of bioluminescence is in high sensitivity, since the signal is detected without background noise, which enables visualization of small tumor burdens and metastases in tissue depth (6, 55). Secondly, as luciferase enzymatic reaction is ATP-dependent, bioluminescence reports the quantity of viable cells (57). However, bioluminescence imaging requires the exogenous luciferin to be administered into mice, thus limiting the repeated image acquisition by the substrate availability. Besides, the commercial systems for bioluminescence imaging allow only detection at the macro scale, i.e., with the mm resolution. Unlike bioluminescence, fluorescence imaging using the genetically encoded fluorescent proteins does not require the injection of a substrate, but it has lower sensitivity due to the presence of the background autofluorescence (58, 59). Visualization of the mKate2 fluorescence signal in our study required the opening of the mouse scalp every time before the imaging procedure. Meanwhile, fluorescence imaging systems, for both microscopic and whole-body imaging, are highly demanded and widely available.

In brain tumor studies, both bioluminescent and fluorescent proteins are equally used. Orthotopic glioma models based on standard cell lines (e.g., U87) genetically transduced with bioluminescent (55, 56) or fluorescent (54, 58, 59) reporter are in routine practice. However, the examples of labeled PDXs are relatively rare (12–15). In our study, labeling of patient-derived glioma cells by both bioluminescent and red fluorescent reporters was successfully realized for the first time, which expanded the choice of methods available for *in vivo* monitoring of tumor development.

To optimize the extent of tumor resection, neurosurgery needs precise tools for intraoperative identification of the tumor. Among available techniques, optical imaging has the advantages of high sensitivity, high spatial resolution, non-invasiveness, low cost, and simplicity of the equipment and, therefore, overcomes limitations of the standard techniques, such as intraoperative MRI or ultrasound. In the last years, the focus of the developments in this field is on label-free technologies that provide endogenous optical contrast of the tumor using its specific morphology or biochemical composition. There are several optical imaging modalities that show remarkable promise to discriminate brain tumors from normal tissue on a label-free basis—OCT, fluorescence-based techniques (e.g., two-photon excited fluorescence microscopy, fiber-based fluorescence spectroscopy, and macroimaging), Raman and coherent anti-Stokes Raman scattering (CARS) microscopy, and spectroscopy (16, 20). For future clinical application, studies on patient-specific models and patient samples are required.

Macro-FLIM was tested on the new GBM model. Previously, we have shown on rat glioma models that time-resolved fluorescence in the NAD(P)H spectral range is capable of distinguishing between the tumors and intact brain tissue (24). Differentiation of the tumors from the tumor-distant cortex and the white matter was not always possible due to specifics of the rat tumor’s growth, which had a large size and might affect the properties of normal tissues located at a distance from the tumor. Preliminary results on samples from patients have also demonstrated differences of GBM from the white matter (24). Our present results on PDXs and U87 MG confirmed the possibilities of macro-FLIM for reliable differentiation between the tumors and the white matter in the NAD(P)H spectral channel. Interestingly, some PDXs (4 of 10) showed differences also from the cortex. High inter-tumor heterogeneity of the PDXs generated from the same cell culture could be attributed to a high clonal diversity of the patient’s GBM. It is known that during long-term passaging the overall number of cell clones decreases (60), which explains the uniformity of U87 MG tumors in terms of the autofluorescence lifetime parameters. The presence of metabolically/biochemically distinct samples in the group of PDXs deserves a deeper investigation as it is fundamental for tumor progression. Several previous papers show a shorter NAD(P)H fluorescence lifetime in the tumors of different types compared to normal tissues, which is usually attributed to the Warburg effect (21–23). Although a specific metabolism is inherent also to glioblastoma, interpretation of the differences in fluorescence lifetimes between glioblastoma and normal brain is more complex and should take into account different biochemical compositions of the brain tissues. Specifically, lipids in the myelin sheaths or lipofuscin can contribute to the fluorescence in the blue range along with NAD(P)H (61).

OCT is a promising method of intraoperative navigation in brain glioma surgery (28–30). The OCT data can be analyzed qualitatively (62–64) and quantitatively (26, 27, 64), but preference is generally given to color-coding of the OCT signal using the attenuation coefficients (26–28). In this paper, we used OCT/CP OCT to visualize GBM7-Luc2-mKate2 PDXs in mouse brain and found that tumor can be differentiated from the normal white matter using optical coefficients. This result is in agreement with our previous data obtained on rat glioma model (29) and patient glioma samples (28). Although the OCT criteria for the separation of tumorous and normal brain tissues were suggested (26, 28), the studies on patient samples are limited. In the newly created human GBM xenograft, areas of invasive growth with a high density of tumor cells can be identified more clearly than in standard rat and mouse glioma models. We believe that the novel tumor model will help to investigate tumor invasion patterns of cancer invasion in more detail, including the degree of preserved myelinated fibers in the perifocal zone and mapping the tracts, and to develop design of more robust algorithms for the quantitative analysis evaluation of OCT/CP OCT data.

Therefore, many aspects of glioma growth can be studied using the dual-labeled GBM xenograft and the optical methods macro-FLIM and OCT/CP OCT. Our findings indicate that both innovative optical techniques are suitable for differentiation GBM from normal tissue, which highlights the importance of further clinical investigations of these technologies.

## Conclusions

The study, presented here, established a new intracranial model of GBM on the basis of patient-derived cells dual-labeled with genetically encoded optical reporters. The obtained model possesses several advantages, such as easy maintenance and expansion of tumor cells *in vitro* before their inoculation into a mouse brain, formation of reproducible tumors with reliable rates of growth in nude mice, the possibility to monitor the growth with fluorescence and bioluminescence imaging, and preservation of major molecular markers and histopathological features of patient’s GBM. Our results on macro-FLIM and CP OCT visualization of the novel GBM model demonstrate the high potential of the new methods for intraoperative differentiation of GBM from the white matter. Furthermore, macro-FLIM suggests a high heterogeneity of a patient tumor resulting in the diversity of metabolic phenotypes. It would be of interest to further investigate the origin of metabolic variations between similar tumors. Animal tumor models that closely recapitulate patient tumors are crucially important for investigations of tumor biology and development of new therapeutic and diagnostic techniques.

## Data Availability Statement

The original contributions presented in the study are included in the article/**Supplementary Material**, further inquiries can be directed to the corresponding author/s.

## Ethics Statement

The studies involving human participants were reviewed and approved by local human research ethics committee Federal Research Clinical Center of the FMBA of Russia (protocol #16 from September 30, 2017). Written informed consent for participation was not required for this study in accordance with the national legislation and the institutional requirements. The animal study was reviewed and approved by Ethics Committee of the Privolzhsky Research Medical University (Nizhny Novgorod, Russia), approval #6 from April 17, 2019.

## Author Contributions

MS, DY, and VB conceived and supervised the study. GY, VB, TB, and DM established the patient-derived GBM cell line. DY, DS, EK, VS, ML, and VD performed the experiments and processed, interpreted, and presented the data. LS and EB performed histological and IHC analyses. DY, MS, EK, and VS wrote and revised the manuscript. All authors contributed to the article and approved the submitted version.

## Funding

The work related to macro-FLIM was supported by the Russian Science Foundation (project № 22-29-01198). The work related to immunofluorescence and intravital confocal microscopy was supported by the Russian Science Foundation (project № 21-74-20110).

## Conflict of Interest

Author VS was employed by Becker&Hickl GmbH.

The remaining authors declare that the research was conducted in the absence of any commercial or financial relationships that could be construed as a potential conflict of interest.

## Publisher’s Note

All claims expressed in this article are solely those of the authors and do not necessarily represent those of their affiliated organizations, or those of the publisher, the editors and the reviewers. Any product that may be evaluated in this article, or claim that may be made by its manufacturer, is not guaranteed or endorsed by the publisher.

## References

[1] Tan AC Ashley DM López GY Malinzak M Friedman HS Khasraw M. Management of Glioblastoma: State of the Art and Future Directions. CA A Cancer J Clin (2020) 70:299–312. doi: 10.3322/caac.21613 32478924

[2] Oronsky B Reid TR Oronsky A Sandhu N Knox SJ. A Review of Newly Diagnosed Glioblastoma. Front Oncol (2021) 10:574012. doi: 10.3389/fonc.2020.574012 33614476 PMC7892469

[3] Hicks WH Bird CE Traylor JI Shi DD El Ahmadieh TY Richardson TE. Contemporary Mouse Models in Glioma Research. Cells (2021) 10:712. doi: 10.3390/cells10030712 33806933 PMC8004772

[4] Haddad AF Young JS Amara D Berger MS Raleigh DR Aghi MK. Mouse Models of Glioblastoma for the Evaluation of Novel Therapeutic Strategies. Neuro-Oncol Adv (2021) 3:vdab100. doi: 10.1093/noajnl/vdab100 PMC8403483 34466804

[5] Golebiewska A Hau A-C Oudin A Stieber D Yabo YA Baus V. Patient-Derived Organoids and Orthotopic Xenografts of Primary and Recurrent Gliomas Represent Relevant Patient Avatars for Precision Oncology. Acta Neuropathol (2020) 140:919–49. doi: 10.1007/s00401-020-02226-7 PMC7666297 33009951

[6] da Hora CC Schweiger MW Wurdinger T Tannous BA. Patient-Derived Glioma Models: From Patients to Dish to Animals. Cells (2019) 8:E1177. doi: 10.3390/cells8101177 31574953 PMC6829406

[7] Patrizii M Bartucci M Pine SR Sabaawy HE. Utility of Glioblastoma Patient-Derived Orthotopic Xenografts in Drug Discovery and Personalized Therapy. Front Oncol (2018) 8:23. doi: 10.3389/fonc.2018.00023 29484285 PMC5816058

[8] Shu Q Wong KK Su JM Adesina AM Yu LT Tsang YTM. Direct Orthotopic Transplantation of Fresh Surgical Specimen Preserves CD133+ Tumor Cells in Clinically Relevant Mouse Models of Medulloblastoma and Glioma. Stem Cells (2008) 26:1414–24. doi: 10.1634/stemcells.2007-1009 18403755

[9] Kim K-M Shim J-K Chang JH Lee J-H Kim S-H Choi J. Failure of a Patient-Derived Xenograft for Brain Tumor Model Prepared by Implantation of Tissue Fragments. Cancer Cell Int (2016) 16:43. doi: 10.1186/s12935-016-0319-0 27293382 PMC4901492

[10] Chokshi CR Savage N Venugopal C Singh SK. A Patient-Derived Xenograft Model of Glioblastoma. STAR Protoc (2020) 1:100179. doi: 10.1016/j.xpro.2020.100179 33377073 PMC7757408

[11] Gamboa CM Jara K Pamarthy S Liu L Aiken R Xiong Z. Generation of Glioblastoma Patient-Derived Organoids and Mouse Brain Orthotopic Xenografts for Drug Screening. STAR Protoc (2021) 2:100345. doi: 10.1016/j.xpro.2021.100345 33665625 PMC7903462

[12] Jarzabek MA Huszthy PC Skaftnesmo KO McCormack E Dicker P Prehn JHM. *In Vivo* Bioluminescence Imaging Validation of a Human Biopsy–Derived Orthotopic Mouse Model of Glioblastoma Multiforme. Mol Imaging (2013) 12:7290. doi: 10.2310/7290.2012.00029 23490442

[13] Hettie KS Teraphongphom NT Ertsey RD Rosenthal EL Chin FT. Targeting Intracranial Patient-Derived Glioblastoma (GBM) With a NIR-I Fluorescent Immunoconjugate for Facilitating its Image-Guided Resection. RSC Adv (2020) 10:42413–22. doi: 10.1039/D0RA07245A PMC7747479 33391732

[14] Chang E Pohling C Natarajan A Witney TH Kaur J Xu L. AshwaMAX and Withaferin A Inhibits Gliomas in Cellular and Murine Orthotopic Models. J Neurooncol (2016) 126:253–64. doi: 10.1007/s11060-015-1972-1 PMC5597337 26650066

[15] Koessinger AL Koessinger D Stevenson K Cloix C Mitchell L Nixon C. Quantitative *In Vivo* Bioluminescence Imaging of Orthotopic Patient-Derived Glioblastoma Xenografts. Sci Rep (2020) 10:15361. doi: 10.1038/s41598-020-72322-x 32958777 PMC7506024

[16] Vasefi F MacKinnon N Farkas DL Kateb B. Review of the Potential of Optical Technologies for Cancer Diagnosis in Neurosurgery: A Step Toward Intraoperative Neurophotonics. Neurophoton (2016) 4:11010. doi: 10.1117/1.NPh.4.1.011010 PMC5184765 28042588

[17] Shcheslavskiy VI Shirmanova MV Dudenkova VV Lukyanov KA Gavrina AI Shumilova AV. Fluorescence Time-Resolved Macroimaging. Opt Lett (2018) 43:3152. doi: 10.1364/OL.43.003152 29957804

[18] Zherdeva V Kazachkina NI Shcheslavskiy V Savitsky AP. Long-Term Fluorescence Lifetime Imaging of a Genetically Encoded Sensor for Caspase-3 Activity in Mouse Tumor Xenografts. J BioMed Opt (2018) 23:1. doi: 10.1117/1.JBO.23.3.035002 29500873

[19] Yashin KS Kiseleva EB Gubarkova EV Moiseev AA Kuznetsov SS Shilyagin PA. Cross-Polarization Optical Coherence Tomography for Brain Tumor Imaging. Front Oncol (2019) 9:201. doi: 10.3389/fonc.2019.00201 31001471 PMC6455095

[20] Lakowicz JR ed. Principles of Fluorescence Spectroscopy. Boston, MA: Springer US (2006). doi: 10.1007/978-0-387-46312-4

[21] Skala MC Riching KM Bird DK Gendron-Fitzpatrick A Eickhoff J Eliceiri KW. In Vivo Multiphoton Fluorescence Lifetime Imaging of Protein-Bound and Free Nicotinamide Adenine Dinucleotide in Normal and Precancerous Epithelia. J BioMed Opt (2007) 12:024014. doi: 10.1117/1.2717503 17477729 PMC2743958

[22] Suarez-Ibarrola R Braun L Pohlmann PF Becker W Bergmann A Gratzke C. Metabolic Imaging of Urothelial Carcinoma by Simultaneous Autofluorescence Lifetime Imaging (FLIM) of NAD(P)H and FAD. Clin Genitour Cancer (2021) 19:e31–6. doi: 10.1016/j.clgc.2020.07.005 32771335

[23] Kolenc OI Quinn KP. Evaluating Cell Metabolism Through Autofluorescence Imaging of NAD(P)H and FAD. Antioxidants Redox Signaling (2019) 30:875–89. doi: 10.1089/ars.2017.7451 PMC6352511 29268621

[24] Lukina M Yashin K Kiseleva EE Alekseeva A Dudenkova V Zagaynova EV. Label-Free Macroscopic Fluorescence Lifetime Imaging of Brain Tumors. Front Oncol (2021) 11:666059. doi: 10.3389/fonc.2021.666059 34109119 PMC8181388

[25] Gelikonov VM Romashov VN Shabanov DV Ksenofontov SY Terpelov DA Shilyagin PA. Cross-Polarization Optical Coherence Tomography With Active Maintenance of the Circular Polarization of a Sounding Wave in a Common Path System. Radiophys Quant El (2018) 60:897–911. doi: 10.1007/s11141-018-9856-9

[26] Kut C Chaichana KL Xi J Raza SM Ye X McVeigh ER. Detection of Human Brain Cancer Infiltration Ex Vivo and *In Vivo* Using Quantitative Optical Coherence Tomography. Sci Transl Med (2015) 7:292ra100. doi: 10.1126/scitranslmed.3010611 PMC4482228 26084803

[27] Yuan W Kut C Liang W Li X. Robust and Fast Characterization of OCT-Based Optical Attenuation Using a Novel Frequency-Domain Algorithm for Brain Cancer Detection. Sci Rep (2017) 7:44909. doi: 10.1038/srep44909 28327613 PMC5361149

[28] Yashin KS Kiseleva EB Moiseev AA Kuznetsov SS Timofeeva LB Pavlova NP. Quantitative Nontumorous and Tumorous Human Brain Tissue Assessment Using Microstructural Co- and Cross-Polarized Optical Coherence Tomography. Sci Rep (2019) 9:2024. doi: 10.1038/s41598-019-38493-y 30765763 PMC6375924

[29] Kiseleva EB Yashin KS Moiseev AA Timofeeva LB Kudelkina VV Alekseeva AI. Optical Coefficients as Tools for Increasing the Optical Coherence Tomography Contrast for Normal Brain Visualization and Glioblastoma Detection. Neurophoton (2019) 6:1. doi: 10.1117/1.NPh.6.3.035003 PMC6630098 31312669

[30] Almasian M Wilk LS Bloemen PR van Leeuwen TG ter Laan M Aalders MCG. Pilot Feasibility Study of *In Vivo* Intraoperative Quantitative Optical Coherence Tomography of Human Brain Tissue During Glioma Resection. J Biophot (2019) 12:e201900037. doi: 10.1002/jbio.201900037 PMC7065626 31245913

[31] World Medical Association Declaration of Helsinki: Ethical Principles for Medical Research Involving Human Subjects. JAMA (2013) 310:2191. doi: 10.1001/jama.2013.281053 24141714

[32] Louis DN Ohgaki H Wiestler OD Cavenee WK Burger PC Jouvet A. The 2007 WHO Classification of Tumours of the Central Nervous System. Acta Neuropathol (2007) 114:97–109. doi: 10.1007/s00401-007-0243-4 17618441 PMC1929165

[33] Kutner RH Zhang X-Y Reiser J. Production, Concentration and Titration of Pseudotyped HIV-1-Based Lentiviral Vectors. Nat Protoc (2009) 4:495–505. doi: 10.1038/nprot.2009.22 19300443

[34] Daukste L Basse B Baguley BC Wall DJN. Mathematical Determination of Cell Population Doubling Times for Multiple Cell Lines. Bull Math Biol (2012) 74:2510–34. doi: 10.1007/s11538-012-9764-7 22914970

[35] Moiseev AA Gelikonov GV Terpelov DA Shilyagin PA Gelikonov VM. Noniterative Method of Reconstruction Optical Coherence Tomography Images With Improved Lateral Resolution in Semitransparent Media. Laser Phys Lett (2013) 10:125601. doi: 10.1088/1612-2011/10/12/125601

[36] Gubarkova EV Moiseev AA Kiseleva EB Vorontsov DA Kuznetsov SS Vorontsov AY. Tissue Optical Properties Estimation From Cross-Polarization OCT Data for Breast Cancer Margin Assessment. Laser Phys Lett (2020) 17:075602. doi: 10.1088/1612-202X/ab9091

[37] Ludwig K Kornblum HI. Molecular Markers in Glioma. J Neurooncol (2017) 134:505–12. doi: 10.1007/s11060-017-2379-y PMC5568999 28233083

[38] Tang X Zuo C Fang P Liu G Qiu Y Huang Y. Targeting Glioblastoma Stem Cells: A Review on Biomarkers, Signal Pathways and Targeted Therapy. Front Oncol (2021) 11:701291. doi: 10.3389/fonc.2021.701291 34307170 PMC8297686

[39] Westermark B Pontén J Hugosson R. Determinants for the Establishment of Permanent Tissue Culture Lines From Human Gliomas. Acta Pathol Microbiol Scand A (1973) 81:791–805. doi: 10.1111/j.1699-0463.1973.tb03573.x 4359449

[40] Pontén J Macintyre EH. Long Term Culture of Normal and Neoplastic Human Glia. Acta Pathol Microbiol Scand (1968) 74:465–86. doi: 10.1111/j.1699-0463.1968.tb03502.x 4313504

[41] Ledur PF Onzi GR Zong H Lenz G. Culture Conditions Defining Glioblastoma Cells Behavior: What is the Impact for Novel Discoveries? Oncotarget (2017) 8:69185–97. doi: 10.18632/oncotarget.20193 PMC5620329 28978189

[42] Torsvik A Stieber D Enger PØ Golebiewska A Molven A Svendsen A. U-251 Revisited: Genetic Drift and Phenotypic Consequences of Long-Term Cultures of Glioblastoma Cells. Cancer Med (2014) 3:812–24. doi: 10.1002/cam4.219 PMC4303149 24810477

[43] Zavjalov EL Razumov IA Gerlinskaya LA Romashchenko AV. *In Vivo* MRI Visualization of U87 Glioblastoma Development Dynamics in the Model of Orthotopic Xenotransplantation to the SCID Mouse. Russ J Genet Appl Res (2016) 6:448–53. doi: 10.1134/S2079059716040225

[44] Bianco J Bastiancich C Joudiou N Gallez B des Rieux A Danhier F. Novel Model of Orthotopic U-87 MG Glioblastoma Resection in Athymic Nude Mice. J Neurosci Methods (2017) 284:96–102. doi: 10.1016/j.jneumeth.2017.04.019 28472680

[45] Burgenske DM Talele S Pokorny JL Mladek AC Bakken KK Carlson BL. Preclinical Modeling in Glioblastoma Patient-Derived Xenograft (GBM PDX) Xenografts to Guide Clinical Development of Lisavanbulin—a Novel Tumor Checkpoint Controller Targeting Microtubules. Neuro-Oncology (2022) 24:384–95. doi: 10.1093/neuonc/noab162 PMC8917401 34232318

[46] Lwin TM Hoffman RM Bouvet M. Advantages of Patient-Derived Orthotopic Mouse Models and Genetic Reporters for Developing Fluorescence-Guided Surgery. J Surg Oncol (2018) 118:253–64. doi: 10.1002/jso.25150 PMC6146062 30080930

[47] Hoffman RM. Patient-Derived Orthotopic Xenografts: Better Mimic of Metastasis Than Subcutaneous Xenografts. Nat Rev Cancer (2015) 15:451–2. doi: 10.1038/nrc3972 26422835

[48] Sordat B Wang WR. Human Colorectal Tumor Xenografts in Nude Mice: Expression of Malignancy. Behring Inst Mitt (1984) 74:291–300. 6477358

[49] Engebraaten O Hjortland GO Hirschberg H Fodstad O. Growth of Precultured Human Glioma Specimens in Nude Rat Brain. J Neurosurg (1999) 90:125–32. doi: 10.3171/jns.1999.90.1.0125 10413165

[50] Ozawa T James CD. Establishing Intracranial Brain Tumor Xenografts With Subsequent Analysis of Tumor Growth and Response to Therapy Using Bioluminescence Imaging. J Vis Exp (2010) 41:1986. doi: 10.3791/1986 PMC3149989 20644517

[51] Brehar FM Ciurea AV Chivu M Zarnescu O Radulescu R Dragu D. The Development of Xenograft Glioblastoma Implants in Nude Mice Brain. J Med Life (2008) 1:275–86. PMC3018968 20108505

[52] Wang H Pan J Yu L Meng L Liu Y Chen X. MicroRNA-16 Inhibits Glioblastoma Growth in Orthotopic Model by Targeting Cyclin D1 and WIP1. Onco Targets Ther (2020) 13:10807–16. doi: 10.2147/OTT.S250369 PMC7591102 33122919

[53] Yu MW Quail DF. Immunotherapy for Glioblastoma: Current Progress and Challenges. Front Immunol (2021) 12:676301. doi: 10.3389/fimmu.2021.676301 34054867 PMC8158294

[54] Yang M Baranov E Jiang P Sun FX Li XM Li L. Whole-Body Optical Imaging of Green Fluorescent Protein-Expressing Tumors and Metastases. Proc Natl Acad Sci U.S.A. (2000) 97:1206–11. doi: 10.1073/pnas.97.3.1206 PMC15570 10655509

[55] Kim W Kang BR Kim HY Cho SM Lee Y-D Kim S. Real-Time Imaging of Glioblastoma Using Bioluminescence in a U-87 MG Xenograft Model Mouse. J Kor Soc Appl Biol Chem (2015) 58:243–8. doi: 10.1007/s13765-015-0037-7

[56] Teng J Hejazi S Hiddingh L Carvalho L de Gooijer MC Wakimoto H. Recycling Drug Screen Repurposes Hydroxyurea as a Sensitizer of Glioblastomas to Temozolomide Targeting De Novo DNA Synthesis, Irrespective of Molecular Subtype. Neuro Oncol (2018) 20:642–54. doi: 10.1093/neuonc/nox198 PMC5892145 29099956

[57] Conway M Xu T Kirkpatrick A Ripp S Sayler G Close D. Real-Time Tracking of Stem Cell Viability, Proliferation, and Differentiation With Autonomous Bioluminescence Imaging. BMC Biol (2020) 18:79. doi: 10.1186/s12915-020-00815-2 32620121 PMC7333384

[58] Momiyama M Suetsugu A Chishima T Bouvet M Endo I Hoffman RM. Subcellular Real-Time Imaging of the Efficacy of Temozolomide on Cancer Cells in the Brain of Live Mice. Anticancer Res (2013) 33:103–6. 23267133

[59] Momiyama M Zhao M Kimura H Tran B Chishima T Bouvet M. Inhibition and Eradication of Human Glioma With Tumor-Targeting Salmonella Typhimurium in an Orthotopic Nude-Mouse Model. Cell Cycle (2012) 11:628–32. doi: 10.4161/cc.11.3.19116 PMC3315098 22274398

[60] Innes JA Lowe AS Fonseca R Aley N El-Hassan T Constantinou M. Phenotyping Clonal Populations of Glioma Stem Cell Reveals a High Degree of Plasticity in Response to Changes of Microenvironment. Lab Invest (2022) 102:172–84. doi: 10.1038/s41374-021-00695-2 PMC8784315 34782726

[61] Croce AC Ferrigno A Di Pasqua LG Berardo C Bottiroli G Vairetti M. NAD(P)H and Flavin Autofluorescence Correlation With ATP in Rat Livers With Different Metabolic Steady-State Conditions. Photochem Photobiol (2017) 93:1519–24. doi: 10.1111/php.12804 28696576

[62] Yashin K Bonsanto MM Achkasova K Zolotova A Wael A-M Kiseleva E. OCT-Guided Surgery for Gliomas: Current Concept and Future Perspectives. Diagnostics (2022) 12:335. doi: 10.3390/diagnostics12020335 35204427 PMC8871129

[63] Hartmann K Stein K-P Neyazi B Sandalcioglu IE. Theranostic Applications of Optical Coherence Tomography in Neurosurgery? Neurosurg Rev (2022) 45:421–7. doi: 10.1007/s10143-021-01599-x PMC8827310 34398385

[64] Böhringer HJ Lankenau E Stellmacher F Reusche E Hüttmann G Giese A. Imaging of Human Brain Tumor Tissue by Near-Infrared Laser Coherence Tomography. Acta Neurochir (2009) 151:507–17. doi: 10.1007/s00701-009-0248-y PMC3085760 19343270

