# Corrigendum: Highly invasive fluorescent/bioluminescent patient-derived orthotopic model of glioblastoma in mice

**DOI:** 10.3389/fonc.2022.1040637

**Published:** 2022-09-29

**Authors:** Diana Yuzhakova, Elena Kiseleva, Marina Shirmanova, Vladislav Shcheslavskiy, Daria Sachkova, Ludmila Snopova, Evgeniya Bederina, Maria Lukina, Varvara Dudenkova, Gaukhar Yusubalieva, Tatyana Belovezhets, Daria Matvienko, Vladimir Baklaushev

**Affiliations:** ^1^ Institute of Experimental Oncology and Biomedical Technologies, Privolzhsky Research Medical University, Nizhny Novgorod, Russia; ^2^ R&D Department, Becker&Hickl GmbH, Berlin, Germany; ^3^ Institute of Biology and Biomedicine, Lobachevsky State University of Nizhny Novgorod, Nizhny Novgorod, Russia; ^4^ Laboratory of Molecular Oncology, Federal Research and Clinical Center of Physical and Chemical Medicine, Moscow, Russia; ^5^ Biomedical Research Center, Federal Research and Clinical Center, Federal Medical and Biological Agency, Moscow, Russia; ^6^ Laboratory of Molecular Mechanisms of Regeneration and Aging, Engelhardt Institute of Molecular Biology, Moscow, Russia; ^7^ Department of Molecular Immunology, Institute of Molecular and Cellular Biology SB RAS, Novosibirsk, Russia

**Keywords:** glioblastoma (GBM), primary cell line, patient-derived xenograft (PDX), fluorescence imaging, FLIM (fluorescence lifetime imaging microscopy)

In the published article, there was an error in the order for **Figure 7** and **Figure 8** as published. The images from **Figure 7** and **Figure 8** were interchanged, while the Figure legends were in the right places. The corrected **Figure 7** and **Figure 8** appear below.

**Figure 7:**
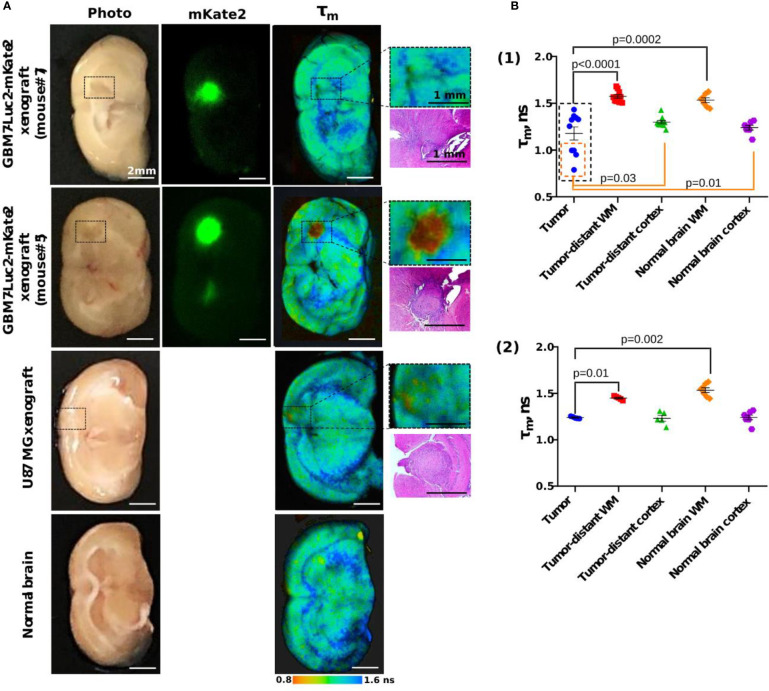
Macro-FLIM of human GBM xenografts and normal brain. **(A)** Representative autofluorescence time-resolved images of GBM7-Luc2-mKate2 xenografts, U87 MG xenograft and normal mouse brain without tumor. Enlarged regions with a tumor are indicated by the black squares on the lower-magnification panel. Corresponding H&E stained section is presented under each enlarged region. **(B)** Quantification of the mean fluorescence lifetime tm in NAD(P)H spectral channel in (1) dual-labeled human GBM xenografts and (2) U87 MG xenografts and normal brain. Scatter dot plot displays the measurements for individual animals (dots) and the mean and SEM (horizontal lines). WM is a white matter.

**Figure 8:**
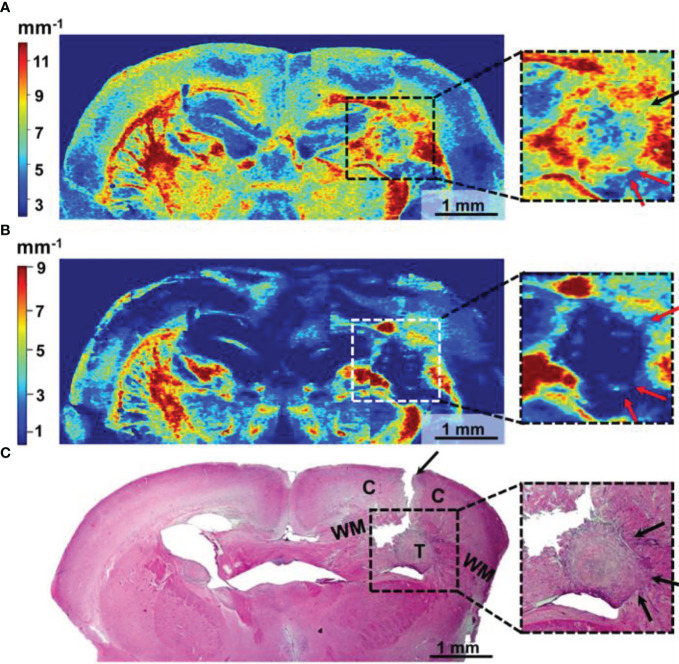
Wide-field OCT color-coded maps of the mouse brain with GBM7-Luc2-mKate2 tumor **(A, B)** and corresponding histology **(C)**. Color-coded maps based on two optical coefficients calculation: attenuation in co-channel (Att_co-_) **(A)** and in cross-channel (Att_cross-_) **(B)**. Perifocal areas of high cancer density are marked with arrows (see enlarged fragments). T, tumor; C, cortex; WM, white matter.

The authors apologize for this error and state that this does not change the scientific conclusions of the article in any way.

## Publisher’s note

All claims expressed in this article are solely those of the authors and do not necessarily represent those of their affiliated organizations, or those of the publisher, the editors and the reviewers. Any product that may be evaluated in this article, or claim that may be made by its manufacturer, is not guaranteed or endorsed by the publisher.

