# Supplementary material for: Highly Invasive Fluorescent/Bioluminescent Patient-Derived Orthotopic Model of Glioblastoma in Mice
Source: Front Oncol. 2022 Jul 13;12:897839. doi: 10.3389/fonc.2022.897839 (PMC9326400; doi:10.3389/fonc.2022.897839)
Supplement: Supplementary file 1 [file DataSheet_1.docx]

Supplementary Material

# Supplementary Data

## Immunohistochemical (IHC) characterization of a patient tumor

Glioblastoma (GBM) specimen has been obtained from the patient (42 years, female) during the surgical resection of left parietal recurrent tumor. Tumor tissue was examined with IHC staining. Moderate diffuse expression of glial fibrillary acidic protein (GFAP) and strong diffuse S100 and p53 expression was observed. In some cells the nuclei were positively stained with c-myc antibodies. The small cell component was diffusely positively stained with CD99 and synaptophysin antibodies. High expression of neuron-specific enolase (NSE) was observed. Proliferation index estimated with Ki67 staining was more than 10% (40-45%). The clinical diagnosis was Glioblastoma (WHO Grade IV) with a primitive neuronal component.

# Supplementary Figures and Tables

## Supplementary Figures


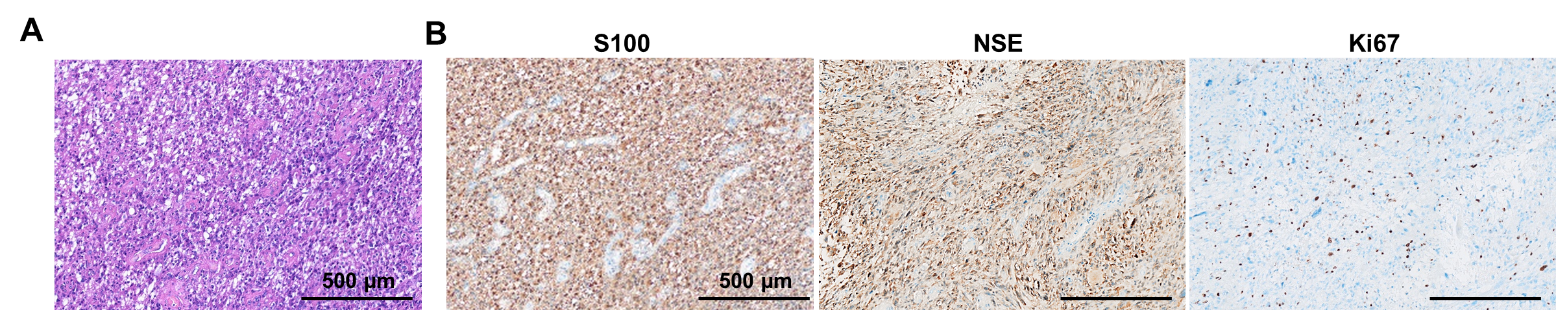


**Supplementary Figure 1**. IHC characterization of the patient GBM sample. Representative H&E (A) and corresponding IHC (B) stained sections. Bars are applicable to all images in the row.

**
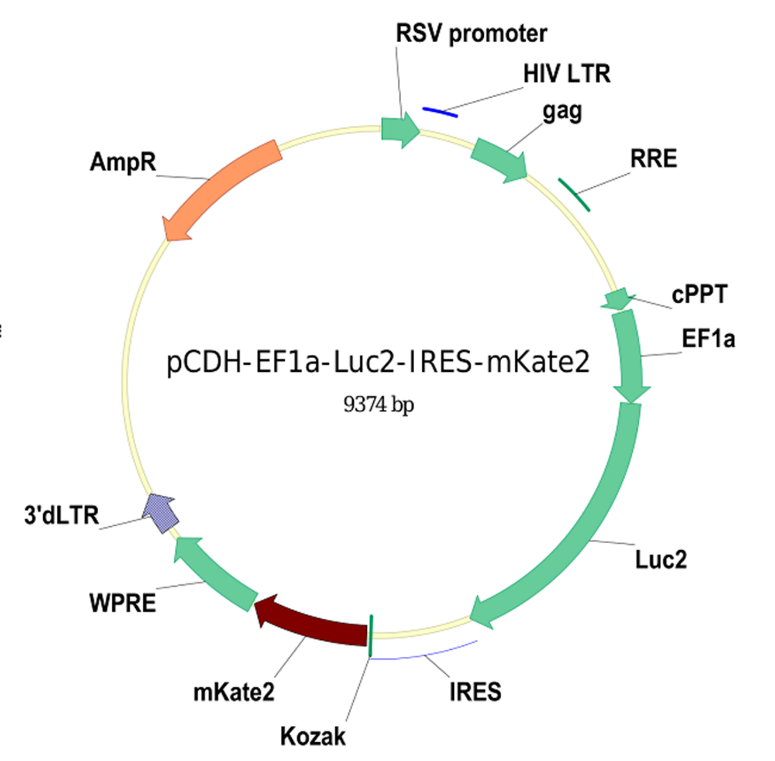
**

**Supplementary Figure 2.** A map of the lentiviral vector pCDH-Luc2-IRES-mKate2 encoding the far-red fluorescent reporter mKate2 as part of the luciferase 2 (Luc2) bicistronic cassette.


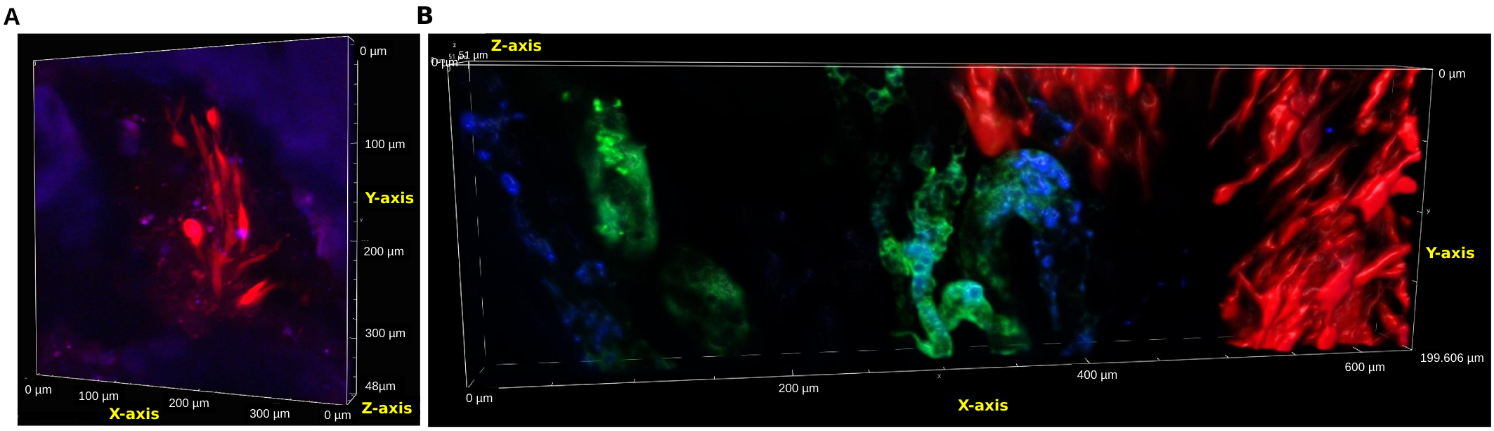


**Supplementary Figure 3.** Intravital microscopy of orthotopic glioblastoma. (**A**) Visualization of mKate2 positive tumor cells in the red channel (Ex560 nm, Em630). (**B**) Imaging of tumor microvessels in the green channel (FITC), Ly6G-positive mononuclear cells in the blue channel, and mKate2-positive glioma cells.


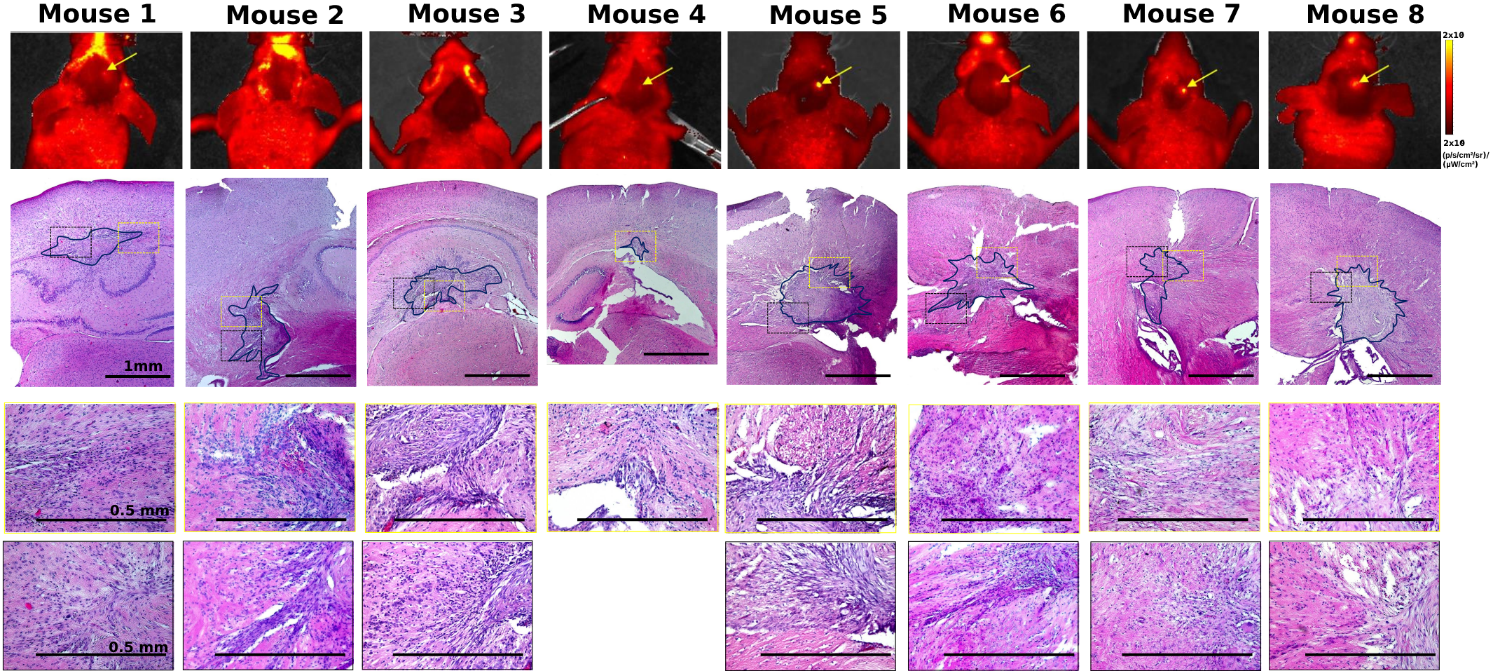


**Supplementary Figure 4.** Verification and characterisation of dual-labeled human GBM xenografts. (**A**) *In vivo* fluorescence imagеs of tumor-bearing mice on 14-15th day after tumor cell inoculation before sacrification. Tumors are indicated by the yellow arrow. (**B**) H&E stained sections of xenograft tumors**.** Enlarged regions are indicated by the yellow and black squares on the lower-magniﬁcation panel. Bars are applicable to all images in the row.

**Table 1**. Autofluorescence lifetimes in GBM, tumor-distant white matter and cortex in dual-labeled human GBM xenografts.

| **Mouse #** |  | ***τ_m_*, ns** | ***τ_1_*, ns** | ***τ_2_*, ns** | ***a_1_*, %** | ***a_2_*, %** |
| --- | --- | --- | --- | --- | --- | --- |
| **1** | **tumor** | 1.35 | 0.58 | 3.47 | 73.57 | 26.43 |
|  | **tumor-distant cortex** | 1.22 | 0.58 | 3.15 | 74.99 | 25.01 |
|  | **tumor-distant white matter** | 1.62 | 0.69 | 3.86 | 70.62 | 29.38 |
| **2** | **tumor** | 1.00 | 0.44 | 3.07 | 78.72 | 21.28 |
|  | **tumor-distant cortex** | 1.29 | 0.65 | 3.21 | 74.55 | 25.45 |
|  | **tumor-distant white matter** | 1.57 | 0.75 | 3.61 | 70.88 | 29.12 |
| **3** | **tumor** | 0.95 | 0.42 | 2.89 | 78.53 | 21.47 |
|  | **tumor-distant cortex** | 1.31 | 0.66 | 3.39 | 76.01 | 23.99 |
|  | **tumor-distant white matter** | 1.52 | 0.72 | 3.61 | 72 | 28 |
| **4** | **tumor** | 1.33 | 0.59 | 3.76 | 76.72 | 23.28 |
|  | **tumor-distant cortex** | 1.43 | 0.74 | 3.77 | 77.15 | 22.85 |
|  | **tumor-distant white matter** | 1.68 | 0.80 | 4.16 | 73.71 | 26.29 |
| **5** | **tumor** | 1.00 | 0.44 | 3.07 | 78.72 | 21.28 |
|  | **tumor-distant cortex** | 1.29 | 0.65 | 3.21 | 74.55 | 25.45 |
|  | **tumor-distant white matter** | 1.57 | 0.75 | 3.61 | 70.88 | 29.12 |
| **6** | **tumor** | 0.79 | 0.34 | 2.66 | 80.61 | 19.39 |
|  | **tumor-distant cortex** | 1.27 | 0.63 | 3.41 | 76.47 | 23.53 |
|  | **tumor-distant white matter** | 1.53 | 0.70 | 3.9 | 73.88 | 26.12 |
| **7** | **tumor** | 1.36 | 0.62 | 3.19 | 70.56 | 29.44 |
|  | **tumor-distant cortex** | 1.28 | 0.66 | 3.28 | 76.2 | 23.8 |
|  | **tumor-distant white matter** | 1.51 | 0.71 | 3.41 | 70.12 | 29.88 |
| **8** | **tumor** | 1.43 | 0.67 | 3.61 | 73.85 | 26.15 |
|  | **tumor-distant cortex** | 1.30 | 0.63 | 3.35 | 75.16 | 24.84 |
|  | **tumor-distant white matter** | 1.51 | 0.72 | 3.68 | 72.83 | 27.17 |
| **9** | **tumor** | 1.32 | 0.61 | 3.24 | 72.53 | 27.47 |
|  | **tumor-distant cortex** | 1.35 | 0.61 | 3.28 | 72.3 | 27.7 |
|  | **tumor-distant white matter** | 1.57 | 0.66 | 3.48 | 67.33 | 32.67 |
| **10** | **tumor** | 1.26 | 0.50 | 3.00 | 69.7 | 30.3 |
|  | **tumor-distant cortex** | 1.27 | 0.56 | 3.33 | 73.72 | 26.28 |
|  | **tumor-distant white matter** | 1.66 | 0.68 | 4.40 | 69.18 | 30.82 |
